# Catalytic hydrogenation of alkyne with planar tetracoordinate carbon in CAl_3_MgH_2_^¯^ system

**DOI:** 10.3389/fchem.2025.1672968

**Published:** 2025-10-31

**Authors:** Abdul Hamid Malhan, Krishnan Thirumoorthy

**Affiliations:** 1 Department of Chemistry, School of Advanced Sciences, Vellore Institute of Technology, Vellore, Tamil Nadu, India; 2 School of Computer Science and Engineering, Vellore Institute of Technology, Vellore, Tamil Nadu, India

**Keywords:** alkyne hydrogenation, planar tetracoordinate carbon, CAl_3_MgH_2_^−^, density functional theory, 2-butyne, 2-butene

## Abstract

The present work reports the catalytic function of the planar tetracoordinate carbon (ptC) molecule, CAl_3_MgH_2_¯, for the first time. The hydrogenation of alkyne and alkene using CAl_3_MgH_2_¯ as a catalyst has been computationally examined through density functional theory calculations. Various quantum chemical tools are employed to analyze the reaction pathways systematically. The study also highlights that the reaction is favourable in the gas phase as compared to the solvent phase, suggesting the practical feasibility of using the CAl_3_MgH_2_^−^ catalyst in the industry. Intrinsic reaction coordinate analysis confirms that the transition states are truly connected to the local minima. Furthermore, natural atomic charges and elongated bond lengths confirm the heterolytic cleavage of H_2_. Non-covalent interaction analysis illustrates the significant role of van der Waals interactions in coordinating reactants and stabilizing products. This study highlights the potential of the ptC molecule CAl_3_MgH_2_^−^ as a catalyst for hydrogenation reactions, eventually opening up new avenues for planar hypercoordinate and main-group metal-based catalysts.

## Introduction

The research on developing an environmentally friendly catalyst for organic transformations is currently a highly active area of focus. One of the most essential and valuable transformations in organic synthesis over the past century has been the hydrogenation of multiple C-C bonds with the addition of H_2,_ and this transformation has widespread applications in various industries ranging from pharmaceuticals to petrochemicals, food, and agriculture (Cano et al., 2021; de Vries and Elsevier, 2007; Johnson et al., 2007; Meijer et al., 2022; Weissermel and Arpe, 2008). Transition metals such as Ru, Rh, Pd, and Ir have traditionally been the most commonly used catalysts in industrial hydrogenation of unsaturated hydrocarbons due to their efficiency in substrate activation and reaction acceleration as demonstrated by the well-known Wilkinson, Schrock-Osborn, Lindlar, and Crabtree catalysts (Crabtree, 1979; Cui and Burgess, 2005; Lindlar, 1952; Nahra and Cazin, 2021; Osborn et al., 1966; Schrock and Osborn, 1976; Zhou, 2016). Despite the indispensable role of transition metal catalysts in hydrogenation transformations, their high cost and, in some cases, the toxicity of transition-metal compounds have prompted the search for alternative, greener, and cost-effective catalysts. Consequently, main-group metals, which are abundant on the earth and environmentally friendly, have gained increasing interest in recent years as a promising approach for future chemistry in industrial applications (Harder, 2012; Hill et al., 2016; Magre et al., 2022; Revunova and Nikonov, 2015; Roy et al., 2021; Yao et al., 2020).

Among these, the main group metal, magnesium, is one of the most abundant metals in the Earth’s crust. Despite its abundance, the application of magnesium-based catalysts for organic reactions is still relatively unexplored (Magre et al., 2022). In 2010, Bonyhady *et al.* reported the first structurally characterized Mg-hydride complexes (Bonyhady et al., 2010), and since then, many efforts have been made to fully understand magnesium chemistry. The application of magnesium-based catalysts has been demonstrated in various reactions, including hydroamination (Dunne et al., 2010; Zhang et al., 2012), hydrosilylation (Garcia et al., 2019a; 2019b; Rauch et al., 2017), hydroboration (Arrowsmith et al., 2012; Magre et al., 2019; Mukherjee et al., 2014; Szewczyk et al., 2019), hydrogenation (Liang et al., 2022), and dehydrocoupling (Liptrot et al., 2014; Ried et al., 2019; Wirtz et al., 2022). The magnesium-based catalyst has demonstrated a major role in various asymmetric reactions (Wang et al., 2024; Yang et al., 2019), including asymmetric hydroboration (Falconnet et al., 2019), asymmetric hydroalkylation (Ye et al., 2025), and asymmetric thia-Michael addition (Jaszczewska-Adamczak et al., 2024). Utilizing main-group metal compounds as catalysts presents a challenge due to their limited ability to activate H_2_ bonds and high reactivity, resulting in unpredictable side reactions. As a result, developing a stable main-group metal complex with enhanced catalytic activity is crucial in addressing this issue. Alternatively, the use of planar hypercoordinate main group elements with demonstrated stability could offer a potential solution.

Considerably, researchers have taken a keen interest in the study of planar tetracoordinate carbon (ptC) and its applications since its discovery in 1968 by H. J. Monkhorst (Monkhorst, 1968). Over the years, ptC has seen significant theoretical and experimental advancements and has also been applied to planar hypercoordination in main group elements (Das et al., 2023; Inostroza et al., 2023; Leyva-Parra et al., 2021; Sun et al., 2020; 2022; Wang et al., 2023; Yang et al., 2015; Zhang et al., 2021). Experimental identification and theoretical confirmation of various aluminum-carbon ptC clusters, such as CAl_4_^2–^, CAl_4_^¯^, and C_2_Al_5_^¯^ as global minimum energy structures have been achieved (Li et al., 1999; 2000; Tsuruoka et al., 2018; Zhang et al., 2023). Furthermore, experimental identification of C_2_Al_4_^¯^ and C_5_Al_5_^¯^ clusters containing ptC has been realized following their theoretical establishment (Naumkin, 2008; Wu et al., 2011; Zhang et al., 2021; 2023). In 2017, Xu *et al.* reported the hydrogenated global minimum ptC of CAl_4_H^0/–^ experimentally and theoretically (Xu et al., 2017). The above-discussed ptC molecules are experimentally identified by generating clusters in the gas phase utilizing a laser vaporization source, followed by their isolation through time-of-flight mass spectrometry, and analysis of their electronic structure via anion photoelectron spectroscopy. The spectral features observed and their comparison with quantum chemical calculations provide direct evidence for the existence of ptC systems.

Similarly, hydrogenated planar systems of CAl_*n*_Be_*m*_H_*x*_^*q*^ (*n* + *m* = 5, *q* = 0, ±1, *x* = *q* + *m* – 1), Al_2_C_4_H_2_, Si_2_C_5_H_2_ and CBe_2_H_5_^¯^ were also reported (Jin et al., 2024; Malhan et al., 2022; Thirumoorthy et al., 2020; Zhao et al., 2018). Other molecular systems involving magnesium, such as CAl_4_Mg^0/–^ and BAl_4_Mg^−/0/+^ with ptC and planar tetracoordinate boron as their global minimum structure, were also theoretically reported (Job et al., 2021; Khatun et al., 2021). These planar clusters promise potential applications in electronics, optoelectronics, and photovoltaics (Yang et al., 2015). In 2023, it was demonstrated that planar hypercoordinate systems of CSi_2_Li_2_, CBe_5_Li_5_^+^, and CS_3_Li_3_^+^ have reversible hydrogen storage properties with high gravimetric densities (Sarmah et al., 2023) due to their unique planar electronic structures and the presence of electropositive metal centers. In 2025, Zhang *et al.* (Zhang et al., 2025) reported the computational investigation of ptC catalysts, specifically C_2_B_2_Me_2_ and C_2_B_2_ *t*Bu*2*, within thermodynamically stable Au^I^ complexes. Their study explored the catalytic activity of these complexes in allylic acetate rearrangement. It was demonstrated that the ptC catalyst (C_2_B_2_Me_2_)Au^I^ exhibits a significantly lower energy barrier for the allylic acetate rearrangement compared to traditional N-heterocyclic carbene–Au^I^ systems, indicating a more favorable reaction both thermodynamically and kinetically. This enhanced performance is attributed to the unique electronic structure of the ptC, which provides a more advantageous electronic environment for the Au center throughout the catalytic cycle. Furthermore, the planarity and electron delocalization inherent to the ptC are not merely passive features, but actively contribute to the catalytic process, resulting in increased activity.

Motivated by these significant advancements, in 2024, our research group theoretically reported a global minimum ptC of the CAl_3_MgH_2_^¯^ system (Malhan and Thirumoorthy, 2024). The delocalization of electron density stabilizes this structure, and the presence of aromaticity in the system was theoretically characterized and confirmed by using various quantum chemical tools. This ptC system was designed using earth-abundant main group elements such as carbon, aluminum, magnesium, and hydrogen. All atoms in the CAl_3_MgH_2_^¯^ molecule participated in the delocalization of electron density except the terminal hydrogen attached to magnesium, which was also found to be hydridic. This discovery led us to explore the potential of using the CAl_3_MgH_2_^¯^ as a catalyst, thereby expanding the application of ptCs in hydrogenation reactions.

So far, despite the lack of well-established laboratory methods for synthesizing ptC, exploring and examining ptC structures could lead to the uncovering and creation of novel materials with exceptional chemical and physical properties. Given the extensive research in ptC chemistry, it is anticipated that potential methods for synthesizing ptC will emerge in the future. Consequently, the significance of the ptC molecule CAl_3_MgH_2_^¯^, which has demonstrated stability, is mainly elevated, especially in its use as a catalyst, particularly in hydrogenation reactions.

In the present work, the catalytic function of the ptC molecule is reported for the first time. The research is focused on the hydrogenation of multiple C-C bonds using CAl_3_MgH_2_^¯^ as a catalyst. The reaction involves heterolytic cleavage of the H_2_ molecule as reported elsewhere (Aireddy and Ding, 2022; Fiorio et al., 2023; Zeng and Li, 2010). This CAl_3_MgH_2_^¯^ catalyst is effective for alkyne, producing alkene. It also facilitates the hydrogenation of alkenes to alkanes under similar conditions. Significantly, a detailed reaction mechanism has been proposed for the catalytic process of hydrogenation of alkyne and alkene using CAl_3_MgH_2_^¯^ catalyst based on quantum chemical studies.

## Computational methodology

Density Functional Theory (DFT) was employed to investigate the hydrogenation of alkyne and alkene. All DFT calculations were carried out using the ωB97XD/6–311++G (2d,2p) (Chai and Head-Gordon, 2008; Clark et al., 1983; Krishnan et al., 1980) level of theory. The ωB97XD functional incorporates empirical dispersion corrections, which are particularly important for reactions involving significant dispersion interactions. Furthermore, the 6–311++G (2d,2p) basis set includes polarization and diffuse functions, which are essential for reactions such as hydrogenation, where changes in electron density and chemical bonding are significant. All optimized structures underwent harmonic vibrational frequency calculations at ωB97XD/6–311++G (2d,2p) level of theory to confirm whether they represent minima or transition states on the potential energy surface. The identification of transition states was achieved through the Berny algorithm implemented in the Gaussian 16 package (Schlegel, 1982). This requests optimization to a transition state rather than a local minimum. The Berny algorithm identifies a transition state using a quasi-Newton approach to approximate the Hessian matrix (second derivative). It locates a stationary point on the potential energy surface, characterized by its Hessian, which has exactly one negative eigenvalue corresponding to the reaction coordinate. The optimization process is adjusted to ensure the Hessian retains this single negative eigenvalue throughout the search. Subsequently, the algorithm progresses uphill direction to the first-order saddle point (transition state), which is maximum in one direction and minimum in all other directions. The solvent effect of toluene on the quantum chemical system was employed using the self-consistent reaction field (SCRF) method with the polarizable continuum model (PCM) to account for the solvation effects implicitly (Scalmani and Frisch, 2010; Tomasi et al., 2005). This technique implicitly accounts for the solvent by surrounding the solute with a reaction field, treating the surrounding solvent molecules as a continuous medium. The SCRF method considers the electrostatic interactions between the solute and the solvent, influencing the solute’s electronic structure and energy levels. This approach facilitates practical calculations by significantly reducing computational costs while effectively balancing computational efficiency. Following the optimization of the molecular geometry within the PCM solvation environment, a frequency calculation was performed on the optimized solvated structure incorporating a pressure of 5 bar. The specified pressure effect is taken into account during the subsequent statistical thermochemical analysis, where it influences the Gibbs free energy by adjusting the translational entropy component in accordance with the ideal gas law. Various quantum chemical tools were utilized to thoroughly characterize the chemical bonding features and reaction pathways of the hydrogenation of alkyne and alkene. Intrinsic reaction coordinate (Gonzalez and Schlegel, 1989) (IRC) calculations were performed to determine the minimum energy reaction pathway from the optimized transition state structures. Furthermore, natural population analysis (Reed et al., 1985) was executed to obtain natural atomic charges using the Gaussian 16 program (Frisch et al., 2016). Additionally, non-covalent interaction (NCI) analysis was carried out using the Multiwfn 3.8 software (Lu and Chen, 2012). All calculations were conducted at the ωB97XD/6–311++G (2d,2p) level of theory using the Gaussian 16 package (Frisch et al., 2016).

## Results and discussion

**FIGURE 1:**
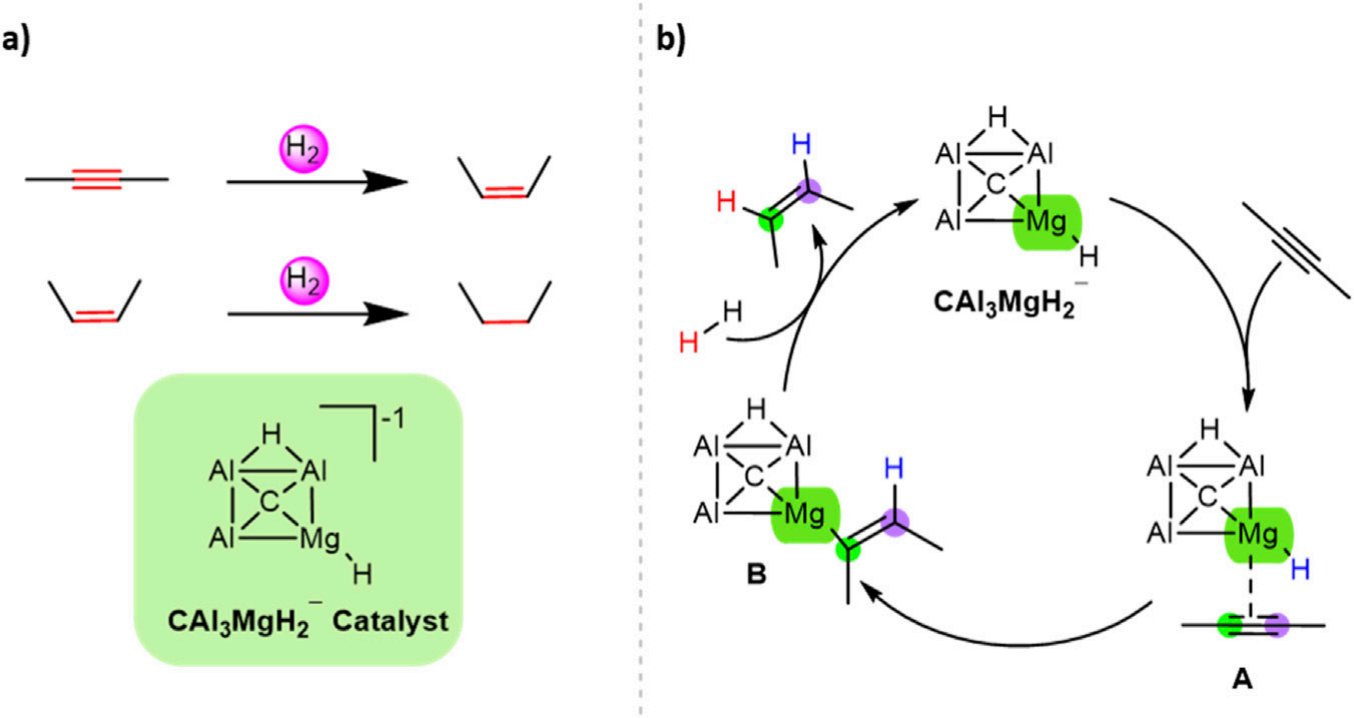
**(a)** Hydrogenation of 2-butyne and 2-butene using CAl_3_MgH_2_^¯^ catalyst. **(b)** Proposed mechanism for hydrogenation of 2-butyne using CAl_3_MgH_2_^¯^ catalyst.

The investigation of the hydrogenation of 2-butyne and 2-butene employing CAl_3_MgH_2_^¯^ as a catalyst is shown in Figure 1a, and the proposed mechanism is illustrated in Figure 1b. In this mechanism, the 2-butyne first coordinates with the magnesium center to form reactant state A. Subsequently, the 2-butyne inserts into the Mg–H bond, leading to an Mg–C bond in B. Then, H_2_ is added to the Mg–C bond to produce the desired product 2-butene, and the catalyst is regenerated.

**FIGURE 2:**
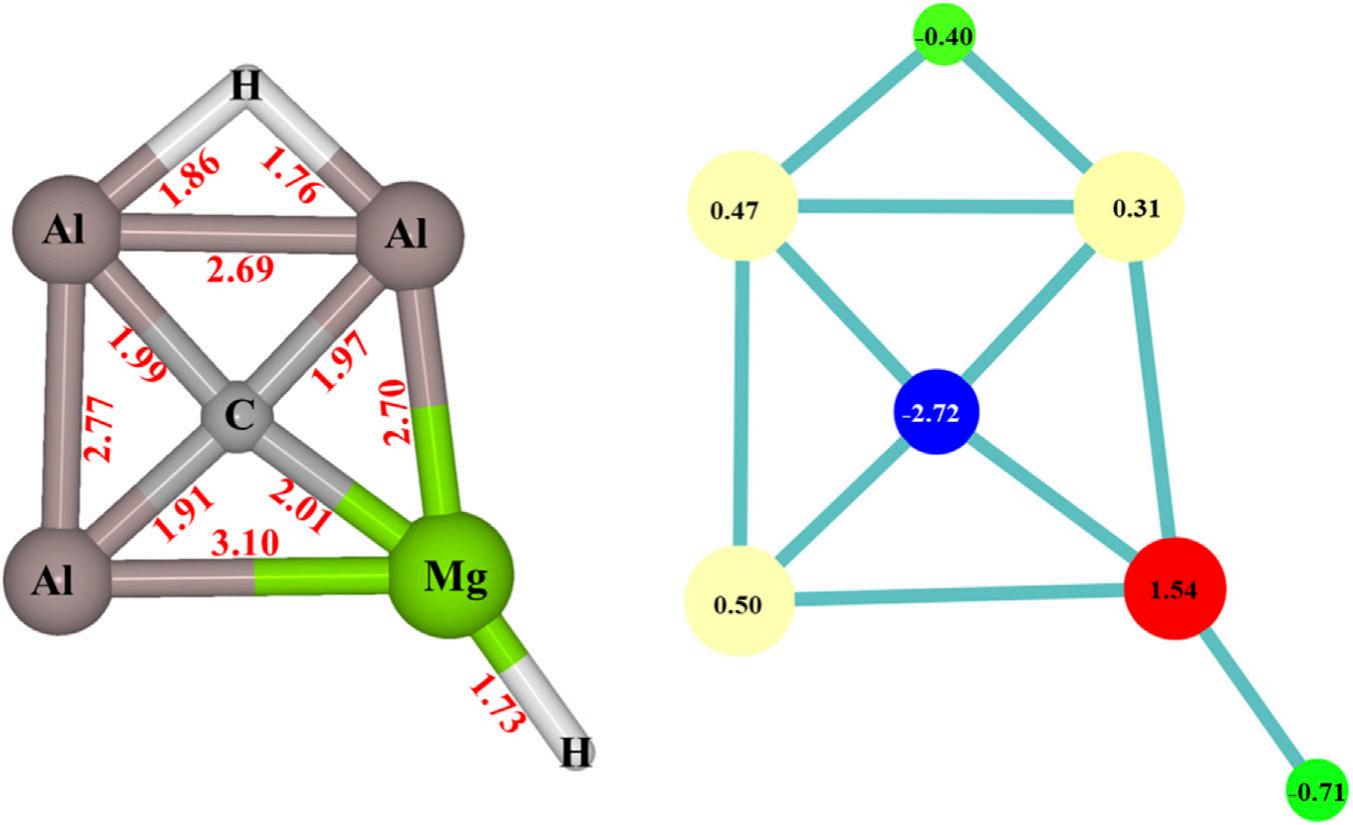
CAl_3_MgH_2_^¯^ with bond lengths (Å) on the left side, and natural atomic charges (|e|) on the right side at ωB97XD/6–311++G (2d,2p) level of theory.

The CAl_3_MgH_2_^¯^ catalyst was meticulously analyzed using various quantum chemical tools, confirming its planar and stable nature, previously reported as a global minimum structure (Malhan and Thirumoorthy, 2024). Figure 2 illustrates the structure of CAl_3_MgH_2_^¯^ along with bond lengths and natural atomic charges. The natural atomic charges from the natural population analysis of bridging and terminal hydride are −0.40 |e| and −0.71 |e|, respectively. The bridging hydride exhibits lower hydridic properties than the terminal hydride and is overlapped with two aluminum atoms, making it challenging for the bridging hydrogen to partake in reactions. On the other hand, the terminal hydride, attached to magnesium with a higher hydridic nature, is more readily available to engage in reactions.

**FIGURE 3:**
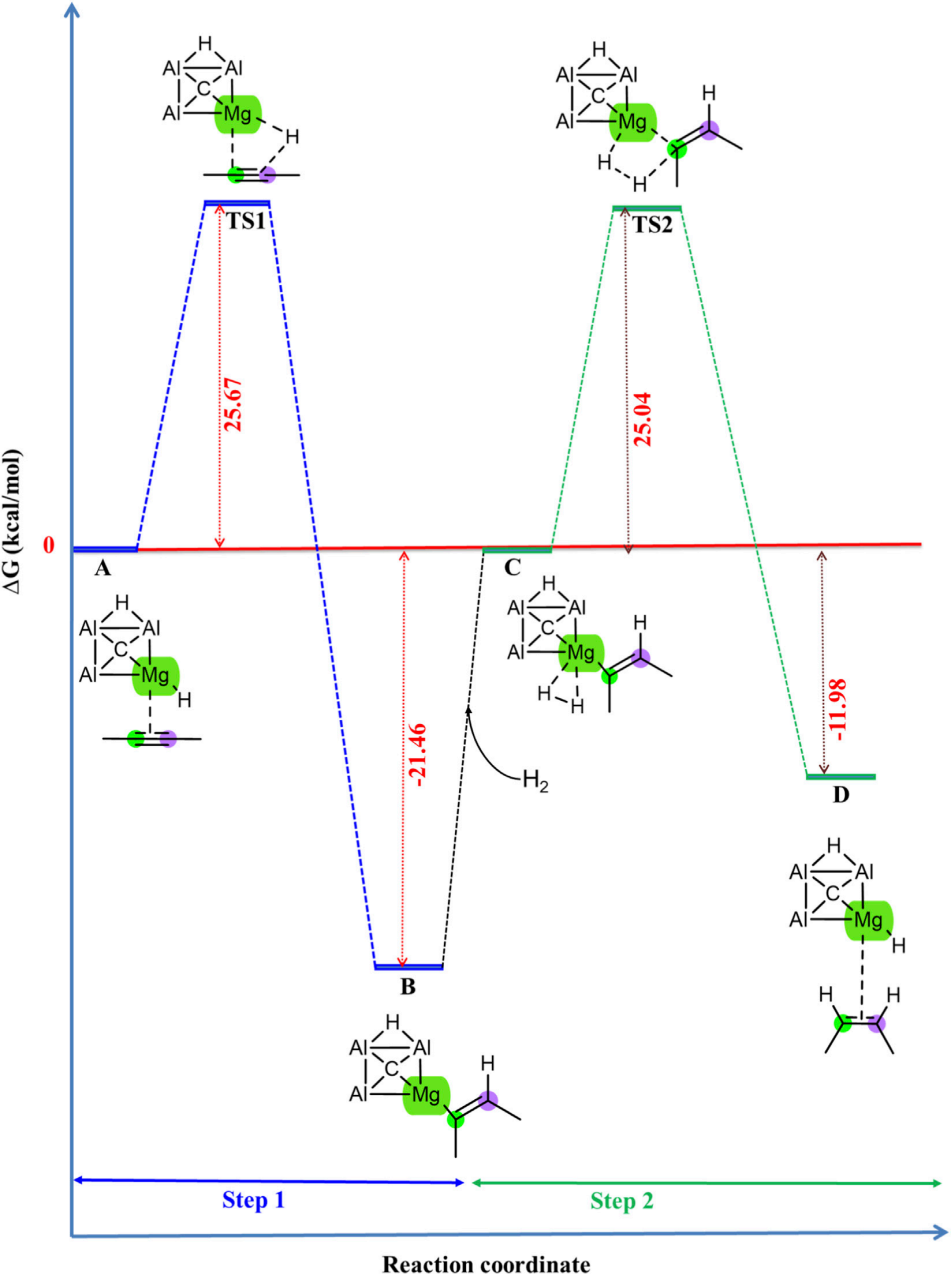
Gibbs free energy profile in kcal/mol of hydrogenation of 2-butyne in the PCM solvation of toluene using CAl_3_MgH_2_^¯^ catalyst. The reaction proceeds via two transition states with activation barriers of 25.67 kcal/mol for TS1 and 25.04 kcal/mol for TS2. All energies are calculated at ωB97XD/6–311++G (2d,2p) level of theory.

The Gibbs free energy profile for the hydrogenation of 2-butyne employing the CAl_3_MgH_2_^¯^ catalyst is given in Figure 3, calculated at the ωB97XD/6–311++G (2d,2p) level of theory, including the PCM solvation of toluene at room temperature (298.15 K) and 5 bar pressure through thermochemical calculations. Additionally, the zero-point corrected energy profile is given in Supplementary Figure S1, the ZPVE corrections, and the number of imaginary frequencies of all the optimized structures of the stationary points involved in the reaction pathway of hydrogenation of 2-butyne using the CAl_3_MgH_2_^¯^ catalyst at the ωB97XD/6–311++G (2d,2p) level of theory in the PCM solvation of toluene are listed in Supplementary Table S1. The transition states, TS1 and TS2, exhibit one imaginary frequency each, confirming that they are first-order saddle points along the reaction pathway. In contrast, structures A, B, C, and D have zero imaginary frequencies, indicating that they are minimum energy structures along the reaction pathway. The reaction commences with the coordination of 2-butyne to the magnesium center of the CAl_3_MgH_2_^¯^ catalyst, forming reactant state A. Subsequently, 2-butyne inserts into the Mg–H bond with an energy barrier of 25.67 kcal/mol, resulting in a stable intermediate B in an exothermic step (−21.46 kcal/mol). Following this, in the second step, H_2_ is added to the Mg–C bond, leading to the cleavage of H_2_ in a heterolytic manner via a “four-center” transition state with an energy barrier of 25.04 kcal/mol, leading to the desired product 2-butene and the regenerated catalyst in D in an exothermic step (−11.98 kcal/mol).

**FIGURE 4:**
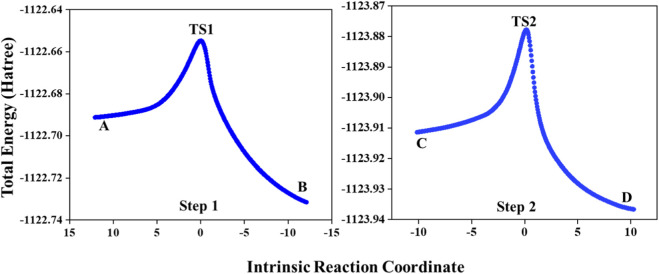
IRC pathway for hydrogenation of 2-butyne in the PCM solvation of toluene using CAl_3_MgH_2_^¯^ catalyst. Both transition states are truly connected to their adjacent local minima. IRC calculations are performed at ωB97XD/6–311++G (2d,2p) level of theory. (IRC video is provided in Supplementary Material).

The identification of transition states was achieved through the Berny algorithm implemented in the Gaussian 16 package, with a specific focus on the hydrogenation of 2-butyne using CAl_3_MgH_2_^¯^. To verify the predicted reaction mechanism of hydrogenation of 2-butyne using CAl_3_MgH_2_^¯^ catalyst, the IRC calculations were conducted as shown in Figure 4. IRC calculation is employed to explore the minimum energy pathways of chemical reactions. It determines the path a system follows from a transition state to connecting the local minima, which corresponds to a stable reactant or product. This process is crucial for understanding the dynamics of chemical reactions, providing an extensive overview of how reactants are systematically converted into products. Both transition state structures, TS1 and TS2, were subjected to IRC calculations, which demonstrate that the transition states are truly connected to the reactants and products. This provides strong validation that the calculated reaction pathway is a minimum energy pathway.

**FIGURE 5:**
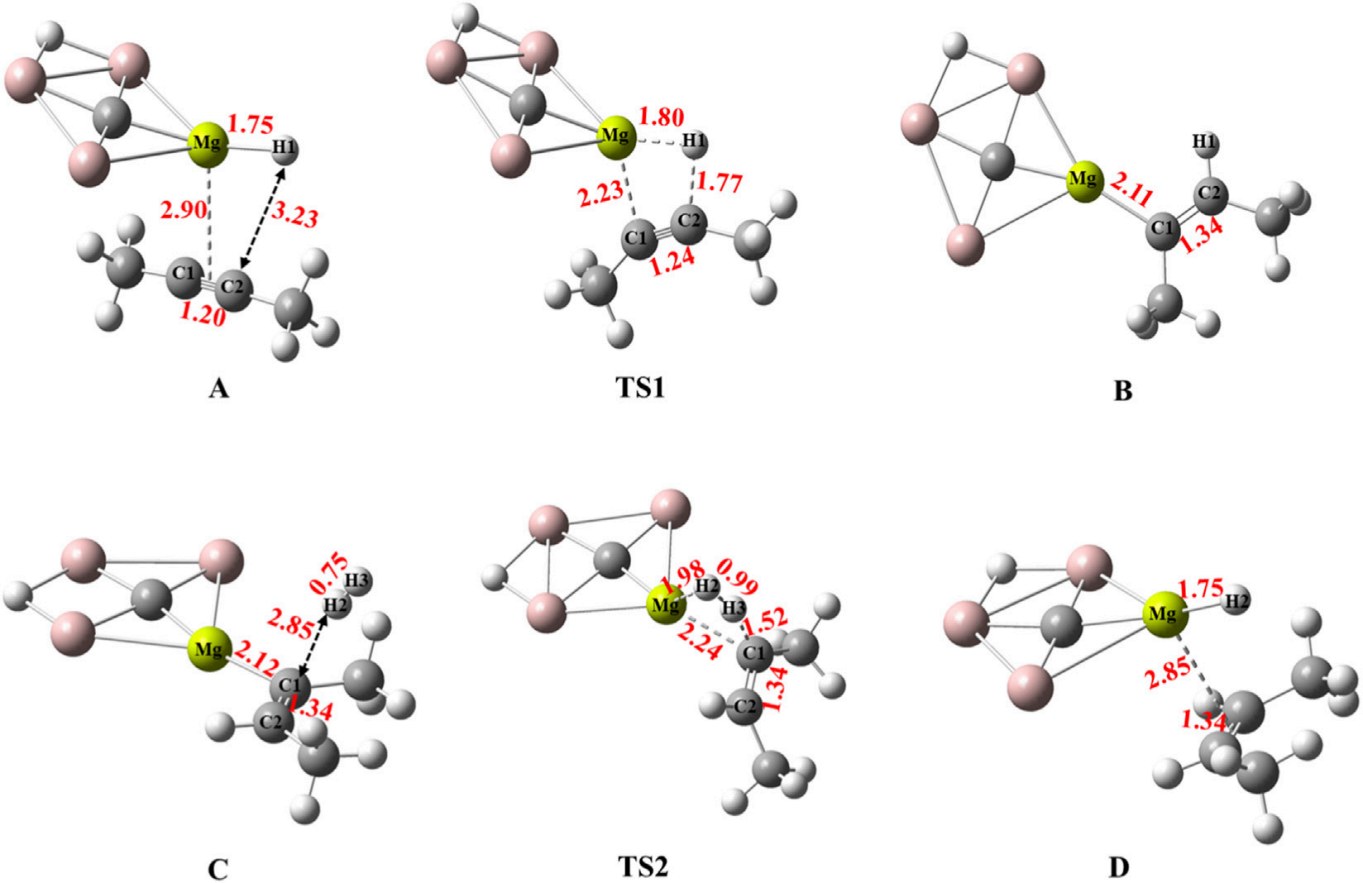
Calculated bond lengths in Å for optimized structures of all the stationary points involved in the reaction pathway of hydrogenation of 2-butyne in the PCM solvation of toluene using CAl_3_MgH_2_^¯^ catalyst at ωB97XD/6–311++G (2d,2p) level of theory. The elongation of the Mg–H1 bond in TS1 confirms the transfer of H1. In TS2, the elongation of H_2_ confirms its cleavage.

The optimized geometries of the stationary points involved in the reaction pathway, including bond lengths, of the hydrogenation of 2-butyne using CAl_3_MgH_2_^¯^ catalyst are presented in Figure 5, accompanied by the corresponding natural atomic charges from natural population analysis as given in Table 1. In reactant state A, the Mg–H1 distance measures 1.75 Å, and the natural atomic charges on Mg and H1 are 1.60 |e| and −0.74 |e|, respectively, indicating the hydridic nature of H1. As 2-butyne approaches the magnesium in TS1, the Mg–H1 bond distance elongates to 1.80 Å, facilitating 2-butyne insertion. In intermediate B, H1 migrates from Mg to carbon (C2), resulting in the loss of its hydridic nature, as evidenced by the natural atomic charges of 0.17 |e| and the formation of a Mg–C1 bond with a distance of 2.11 Å. Moreover, the increase in the C1–C2 bond distance from 1.20 Å to 1.34 Å validates that the triple bond is reduced to a double bond. The natural atomic charges on C1 (−0.62 |e|) indicate that the C1 atom now acts as a Lewis base after bonding to magnesium (1.68|e|). The planar configuration of intermediate B allows the H_2_ molecule to approach from the same side, as in C, which leads to the syn-addition of H_2,_ resulting in the cis-product. In TS2, as the H_2_ molecule approaches the magnesium, the Mg–C1 bond elongates to 2.24 Å. The natural atomic charges on Mg (1.73 |e|) indicate its role as a Lewis acid and on C1 (−0.59 |e|) as a Lewis base. These Lewis acid-base pairs engage in a heterolytic dissociation of H_2_ molecules, breaking them down into proton−hydride pairs, which enhance the overall efficiency of the hydrogenation reaction by facilitating the transfer of protons (H^+^) and hydride ions (H^−^) to the reactants, thereby promoting the formation of new chemical bonds (Aireddy and Ding, 2022). The natural atomic charges of H2 (−0.35 |e|) and H3 (0.09 |e|) in TS2, coupled with the elongation of the H2–H3 bond, confirm the heterolytic cleavage of the H2–H3 bond. Moreover, the bonds Mg−H2 and C1−H3 are being formed, while Mg–C1 and H2–H3 bonds are being broken via a “four-center” transition state in TS2. Finally, the desired product, 2-butene, and the catalyst are regenerated in D.

**TABLE 1:** Natural atomic charges (|e|) on atoms involved in the reaction pathway of hydrogenation of 2-butyne in the PCM solvation of toluene using CAl_3_MgH_2_^¯^ catalyst calculated at ωB97XD/6–311++G (2d,2p) level of theory (Labels are followed as given in Figure 5).

Structure labels	Mg	C1	C2	H1	H2	H3
A	1.60	−0.05	−0.06	−0.74	-	-
TS1	1.72	−0.41	−0.04	−0.48	-	-
B	1.68	−0.62	−0.27	0.17	-	-
TS2	1.73	−0.59	−0.21	0.17	−0.35	0.09
D	1.59	−0.23	−0.23	0.21	−0.74	0.22

**FIGURE 6:**
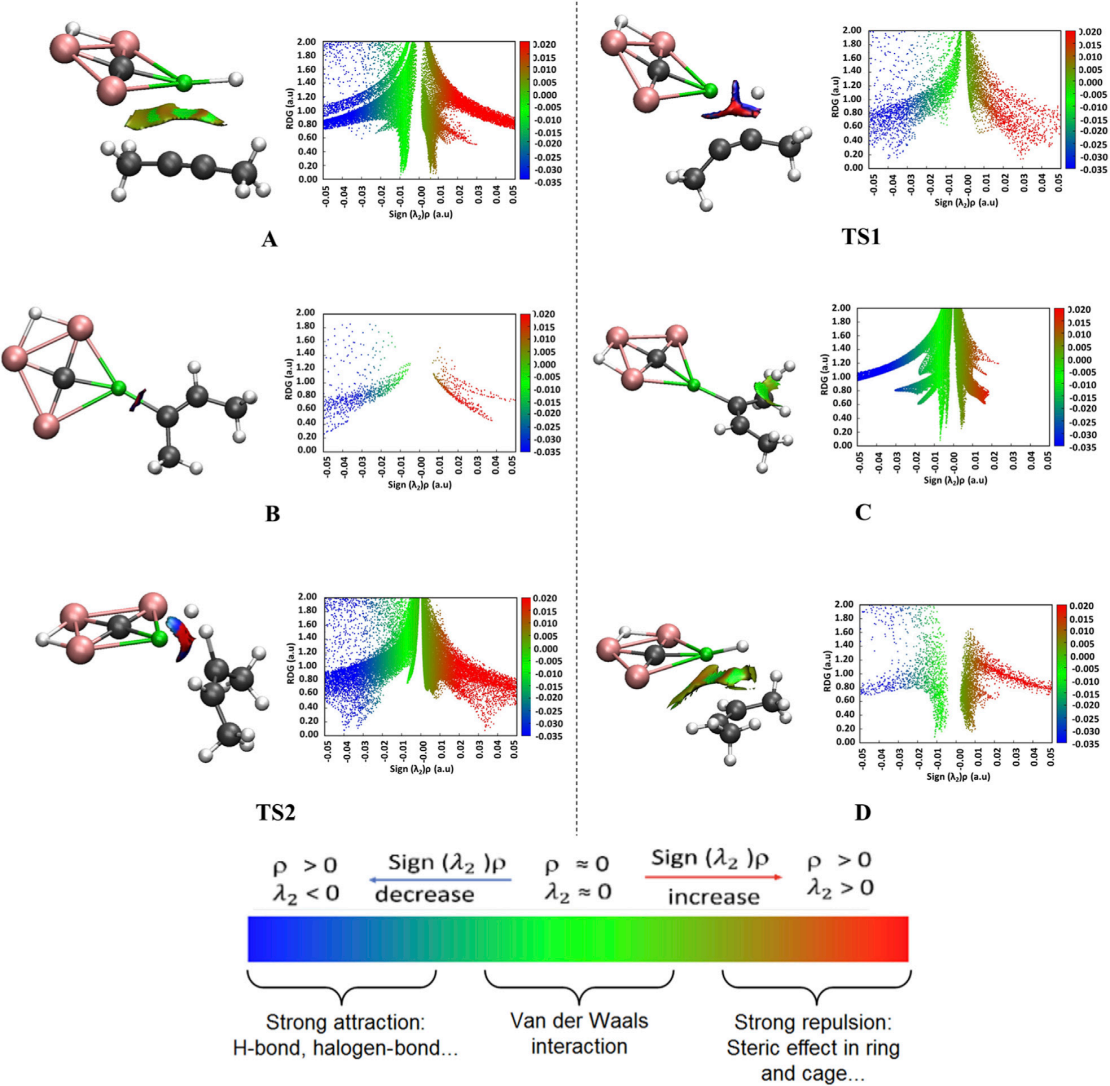
Non-covalent interaction, 3D isosurfaces (on left) and 2D-RDG graphs (on right) for the specific interactions associated with bond breaking and formation involved in the reaction pathway of hydrogenation of 2-butyne in the PCM solvation of toluene using CAl_3_MgH_2_^¯^ catalyst at ωB97XD/6–311++G (2d,2p) level of theory. The reaction initiates with van der Waals interactions (green isosurface) that bring the reactants together in the initial complex. As the system approaches the transition state, these evolve into strong, electrostatic interactions (blue isosurface), which are critical for product formation. Isosurfaces are colored as: strong attraction (blue), van der Waals interaction (green), and repulsive interaction (red). The complete 3D isosurfaces and their corresponding RDG graphs are given in Supplementary Figure S2.

The NCI analysis was conducted using converged Self-Consistent Field (SCF) calculations with cutoff values of 0.5 to gain insights into the type of interactions that happen during the hydrogenation of 2-butyne using the CAl_3_MgH_2_^¯^ catalyst. This involved generating 3D isosurfaces and 2D reduced density gradient (RDG) graphs for all the stationary points along the reaction pathway, as illustrated in Figure 6. This figure specifically highlights the interactions associated with bond breaking and formation. For a comprehensive view, the complete 3D isosurfaces and their corresponding RDG graphs are given in Supplementary Figure S2. The atom labels used here follow as shown in Figure 5. The natural bonding orbital analysis was conducted to understand the orbital interactions that lead to bond formation. The results of this analysis are presented in Supplementary Table S2. It serves to complement the NCI studies through the decomposition of the Wiberg bond order within the framework of natural atomic orbitals. In reactant state A, the green patches represent van der Waals interactions, indicating coordination between the reactants for initiating the reaction process. Upon entering transition state TS1, the blue patches observed between the C2 and H1 atoms indicate a strong attraction stemming from the orbital interaction between 2p_y_ (C2)–1s (H1), exhibiting a contribution of 0.11 (see Supplementary Table S2). These interactions play a crucial role in facilitating bond formation between C2–H1, followed by the formation of the bond between Mg–C1, which leads to the formation of intermediate B. In reactant state C, the H_2_ molecule exhibits van der Waals interactions with the C1–C2 double bond, which leads to the TS2. In TS2, the bonding occurs between C1–H3 (2p_z_ (C1)–1s (H3) contributing 0.19) due to significant orbital interactions. Simultaneously, bond formation takes place between Mg–H2, which is indicated by the blue patches. This process leads to the regeneration of the catalyst and ultimately results in the formation of the desired product in state D. Additionally, in state D, the presence of green patches between the catalyst and 2-butene suggests that the product complex is stabilized through van der Waals interactions.

In 2022, Liang *et al.* conducted both experimental and theoretical studies on the hydrogenation of 2-butyne using a magnesium pincer complex as a catalyst (Liang et al., 2022). The CAl_3_MgH_2_^¯^ may exhibit a similar mechanism to the experimentally reported magnesium pincer catalyst (Liang et al., 2022). Thus, we re-optimized the energy profile of the σ-bond metathesis pathway (similar to the proposed mechanism in the present work, as shown in Figure 1). The Gibbs free energy profile for the hydrogenation of 2-butyne by using the magnesium pincer catalyst is given in Supplementary Figure S3, calculated at the ωB97XD/6–311++G (2d,2p) level of theory, including the PCM solvation of toluene at room temperature (298.15 K) and 5 bar pressure through thermochemical calculations. Additionally, the zero-point corrected energy profile is given in Supplementary Figure S4, the ZPVE corrections, and the number of imaginary frequencies of all the optimized structures of the stationary points involved in the reaction pathway of hydrogenation of 2-butyne using the magnesium pincer catalyst at the ωB97XD/6–311++G (2d,2p) level of theory are listed in Supplementary Table S3. The transition states, TS1 and TS2, exhibit one imaginary frequency each, confirming that they are first-order saddle points along the reaction pathway. In contrast, structures A, B, C, and D have zero imaginary frequencies, indicating that they are minima along the reaction pathway.

**FIGURE 7:**
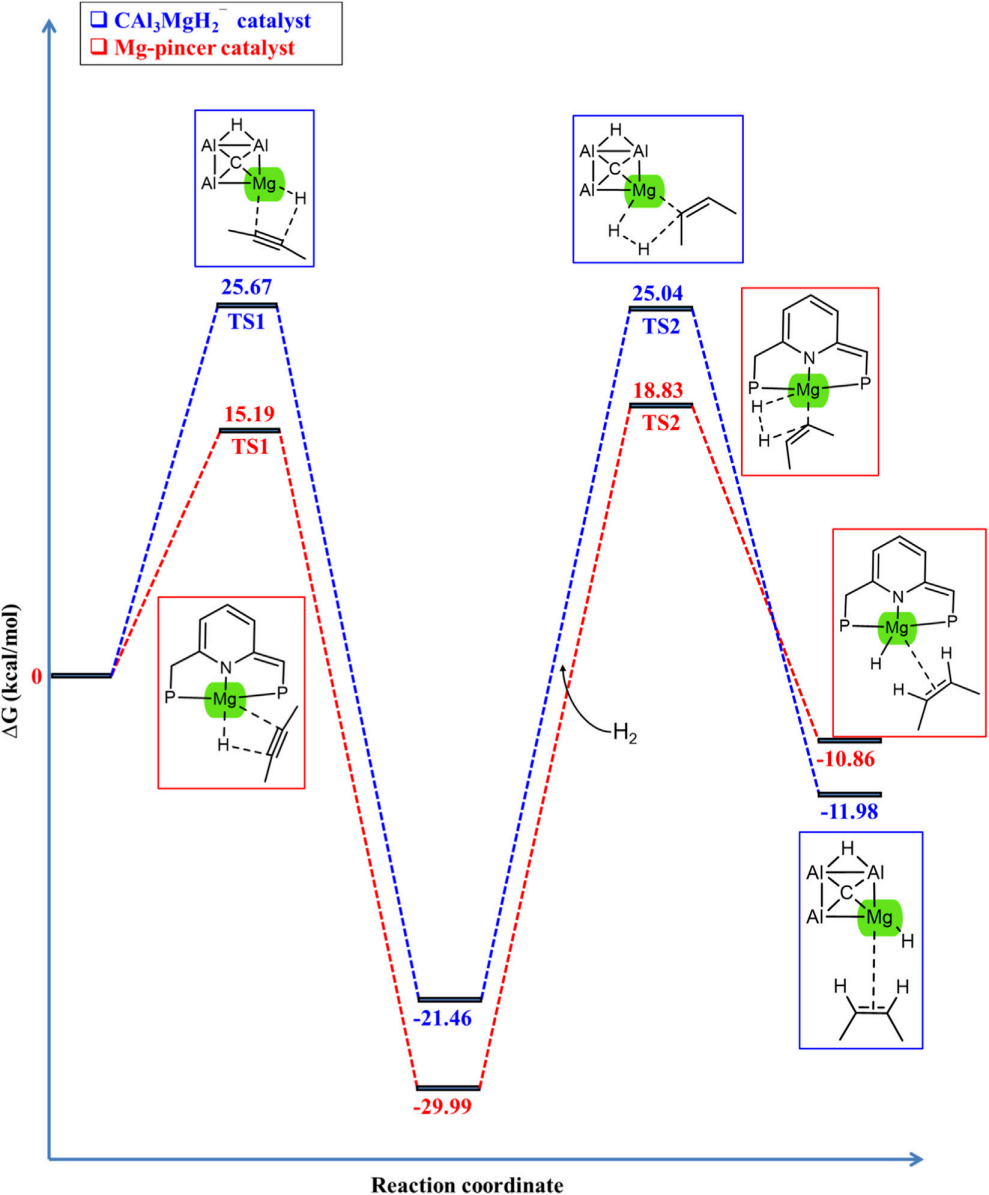
Comparison of Gibbs free energy profile in kcal/mol for hydrogenation of 2-butyne in the PCM solvation of toluene between CAl_3_MgH_2_^¯^ and magnesium pincer catalyst at ωB97XD/6–311++G (2d,2p) level of theory.

This investigation was conducted to establish a comparative analysis with the present study, as the findings indicate that the reaction mechanism involved in the hydrogenation of 2-butyne utilizing a magnesium pincer catalyst is similar to that observed when employing the CAl_3_MgH_2_^¯^ catalyst. The solvent effect of toluene at room temperature with 5 bar pressure for thermochemical calculation was incorporated into the current study to closely replicate the conditions utilized in the experimental investigation of the magnesium pincer catalyst. This approach aims to enable more accurate comparative analyses. To illustrate this, Figure 7 compares the Gibbs free energy profile for the hydrogenation of 2-butyne between the CAl_3_MgH_2_^¯^ and magnesium pincer catalyst, and their energy differences are given in Supplementary Table S4. Additionally, the comparison with the zero-point corrected energy profile is given in Supplementary Figure S5. The energies of transition states TS1 and TS2 using the CAl_3_MgH_2_^¯^ catalyst are higher than those using the magnesium pincer catalyst by 10.48 kcal/mol and 6.21 kcal/mol.

**FIGURE 8:**
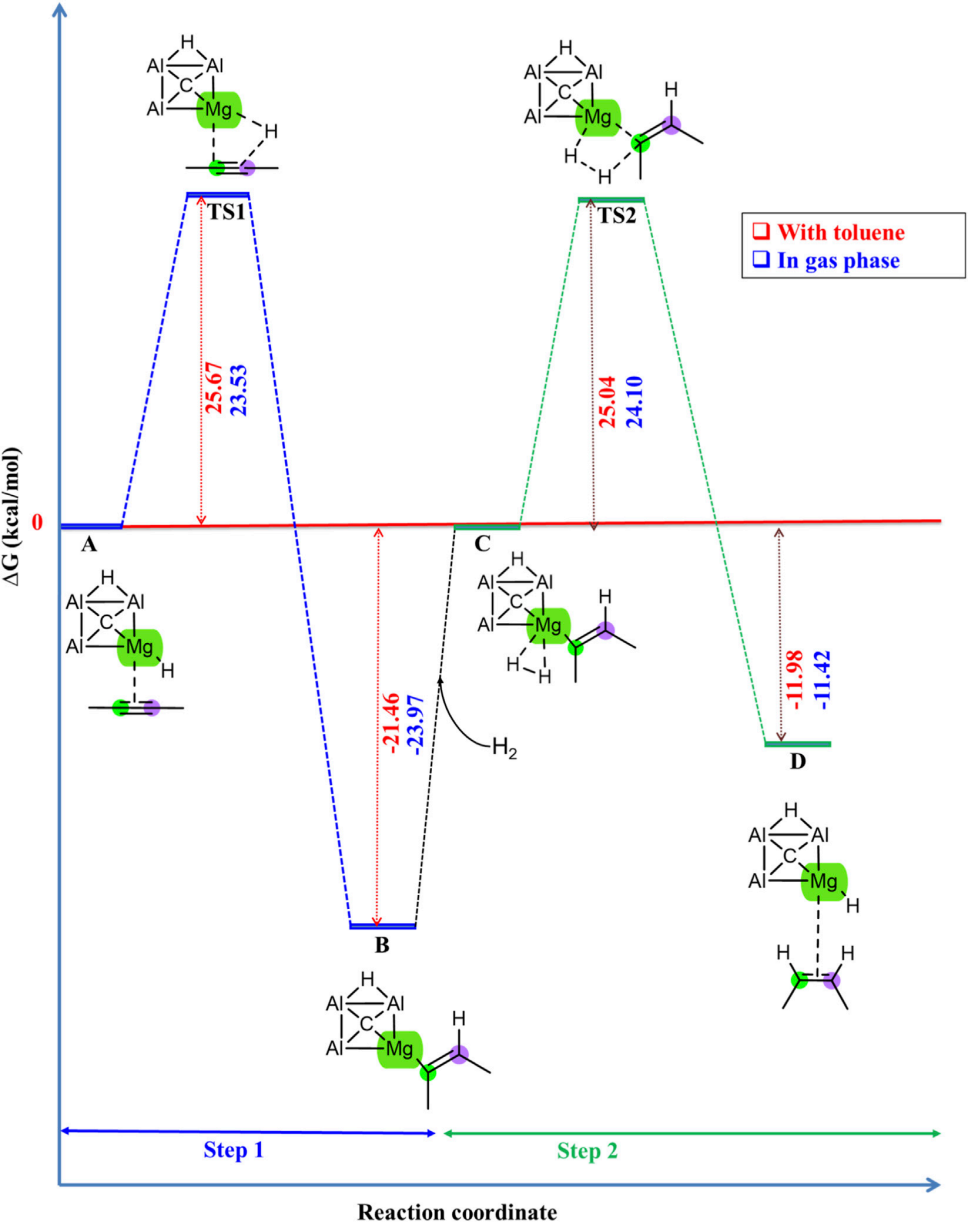
Gibbs free energy profile comparison in kcal/mol for hydrogenation of 2-butyne using CAl_3_MgH_2_^¯^ catalyst in PCM solvation of toluene and gas phase. The lower activation barrier in the gas phase favors the reaction kinetically compared to the solvent phase. All energies are calculated at ωB97XD/6–311++G (2d,2p) level of theory.

The hydrogenation of 2-butyne using CAl_3_MgH_2_^¯^ has been extensively investigated, both in PCM solvation of toluene and in the gas phase. The comparison of their Gibbs free energy profiles is illustrated in Figure 8. Additionally, the comparison with the zero-point corrected energy profile is given in Supplementary Figure S6. The IRC pathway in the gas phase is given in Supplementary Figure S7, which confirms that the transition states are truly connected to their adjacent local minima. In the gas phase, the transition states TS1 and TS2 are found to be energetically favorable, with energy levels lower by 2.14 kcal/mol and 0.94 kcal/mol, respectively, than in the solvent phase. The Zero-point corrected energies and ΔG values provided in Table 2 for comparison between the gas and the solvent phase of toluene further confirm that the gas phase offers a reduced activation barrier for the reaction. Moreover, the CAl_3_MgH_2_^¯^ catalyst demonstrated exceptional catalytic efficiency under gas phase conditions than the solution phase, indicating its promising applications in industrial settings. The optimized geometries involved in the reaction pathway detailing essential bond lengths are displayed in Supplementary Figure S8, and their corresponding natural atomic charges are provided in Supplementary Table S5, offering insights into electron distribution during the reaction. Furthermore, the NCI analysis is given in Supplementary Figure S9, which specifically highlights the interactions associated with bond breaking and formation. For a comprehensive view, the complete 3D isosurfaces and their corresponding RDG graphs are given in Supplementary Figure S10. In reactant state C, with toluene as the solvent, the H_2_ molecule exhibits van der Waals interactions with the C1–C2 double bond, likely attributable to solvent-induced polarization. Conversely, in the gas phase, H_2_ predominantly interacts through van der Waals forces with the Mg atom (see Supplementary Figure S11). This underscores the significant impact of the environment on molecular interactions in computational investigations.

**TABLE 2:** Comparison between ΔG, ΔE + ZPVE, and ΔE energies in kcal/mol for hydrogenation of 2-butyne in both gas phase and in PCM solvation of toluene using CAl_3_MgH_2_^¯^ catalyst. The lower activation barrier in the gas phase favors the reaction kinetically compared to the solvent phase. All energies are calculated at ωB97XD/6–311++G (2d,2p) level of theory.

Structure labels	ΔG (kcal/mol)		ΔE + ZPVE (kcal/mol)		ΔE (kcal/mol)	
	Solvent	Gas	Solvent	Gas	Solvent	Gas
A	0.00	0.00	0.00	0.00	0.00	0.00
TS1	25.67	23.53	24.03	22.84	23.91	23.03
B	−21.46	−23.97	−22.72	−24.03	−26.67	−27.42
C	0.00	0.00	0.00	0.00	0.00	0.00
TS2	25.04	24.09	23.34	22.46	22.78	21.90
D	−11.99	−11.42	−14.18	−13.11	−17.76	−16.75

**FIGURE 9:**
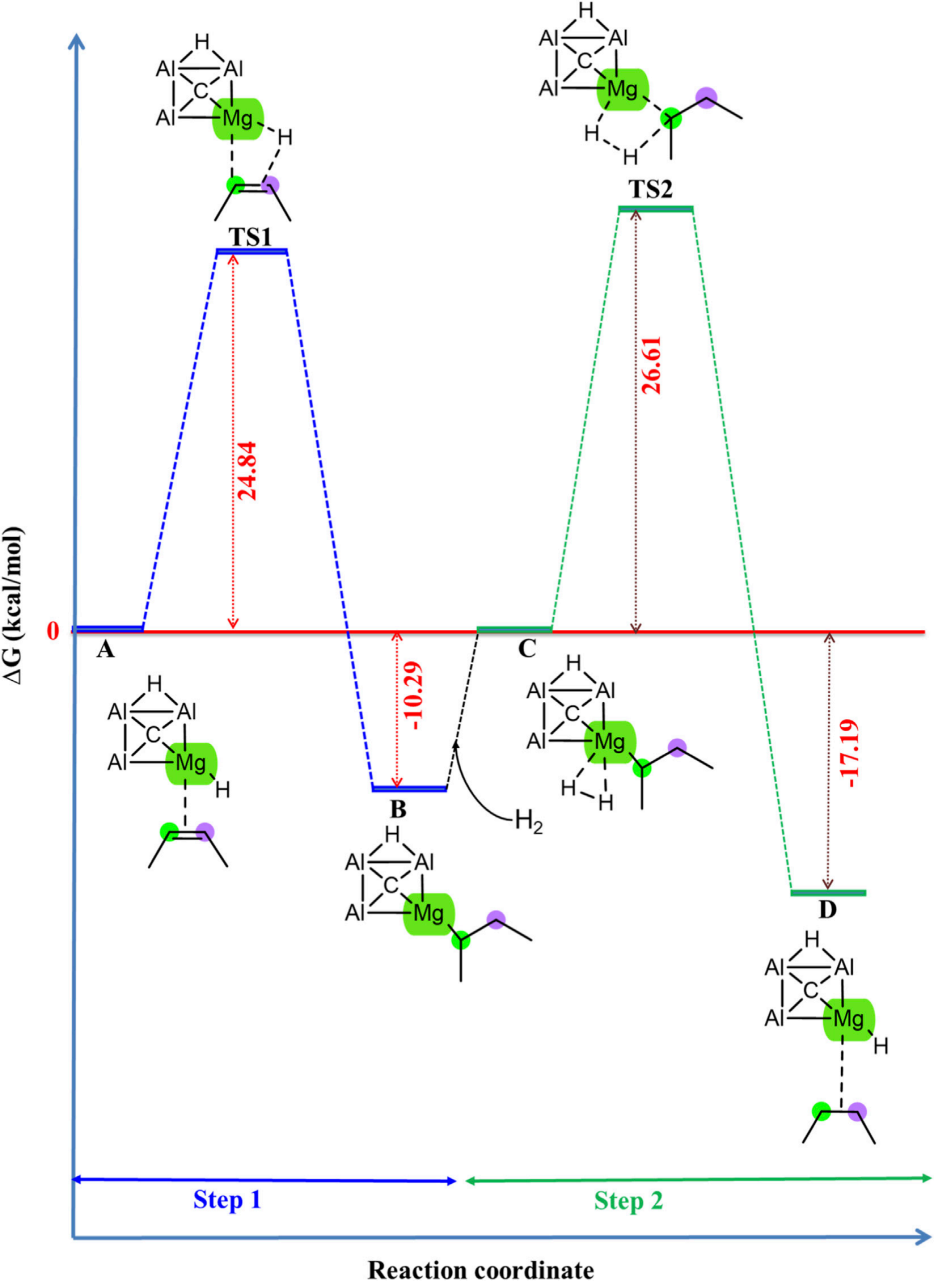
Gibbs free energy profile in kcal/mol of hydrogenation of 2-butene in the PCM solvation of toluene using CAl_3_MgH_2_^¯^ catalyst. The reaction proceeds via two transition states with activation barriers of 24.84 kcal/mol for TS1 and 26.61 kcal/mol for TS2. All energies are calculated at ωB97XD/6–311++G (2d,2p) level of theory.

It is also investigated whether the CAl_3_MgH_2_^¯^ catalyst used in the hydrogenation of 2-butyne is also suitable for the further hydrogenation of 2-butene. Interestingly, the CAl_3_MgH_2_^¯^ catalyst demonstrated promising catalytic efficiency under similar conditions for the hydrogenation of 2-butene, as evidenced by its Gibbs free energy profile in Figure 9. Additionally, the zero-point corrected energy profile is given in Supplementary Figure S12. The IRC pathway is given in Supplementary Figure S13, which confirms that the transition states are truly connected to their adjacent local minima. Its optimized geometries involved in the reaction pathway, including bond lengths, are displayed in Supplementary Figure S14, and the corresponding natural atomic charges are provided in Supplementary Table S6. This observation suggests that the mechanism for the hydrogenation of 2-butene is similar to that for the hydrogenation of 2-butyne using the CAl_3_MgH_2_^¯^ catalyst.

In the previous study, the stability of CAl_3_MgH_2_^¯^ (Malhan and Thirumoorthy, 2024) was computationally characterized and confirmed using various quantum chemical tools. These tools indicated that the stability arises from the delocalization of electron density and the presence of double aromaticity within system. A similar structure, CAl_4_H^¯^ (Xu et al., 2017), has already been identified experimentally. By substituting one aluminum atom with an isoelectronic Mg–H unit in CAl_4_H^¯^, there exists a promising opportunity for the experimental identification of CAl_3_MgH_2_^¯^. Although several ptC have been reported experimentally in the gas phase, well-established laboratory methods for synthesizing ptC are currently lacking. Given the extensive research in the chemistry of ptC, it is expected that potential methods for its synthesis will emerge in the future.

## Conclusion

In summary, the hydrogenation of an alkyne using the CAl_3_MgH_2_^¯^ catalyst has been thoroughly investigated through computational calculations. In this reaction, first, the alkyne inserts into the Mg–H bond, followed by the cleavage of H_2_ in a heterolytic manner, leading to hydrogenation and the regeneration of the catalyst. The comparison of the CAl_3_MgH_2_^¯^ catalyst with the magnesium pincer catalyst indicated that the transition states are higher with the CAl_3_MgH_2_^¯^ catalyst. The comparison between the PCM solvation of toluene and the gas phase using the CAl_3_MgH_2_^¯^ catalyst indicated that the reaction can proceed even under milder conditions. The reaction pathway is also confirmed to be a minimum energy pathway, as evidenced by IRC calculations. Natural atomic charges from natural population analysis confirmed that H_2_ bond activation is facilitated by the Lewis acid-Lewis base (Mg^δ+^–C^δ−^) mechanism. NCI analysis alongside natural atomic orbital demonstrated that van der Waals interactions play a significant role in coordinating reactants and stabilizing products. Furthermore, NCI studies indicated that bond formation and breaking occur simultaneously in the transition state structures, which are also supported by natural bonding orbital analysis. It was also discovered that the CAl_3_MgH_2_^¯^ catalyst could also hydrogenate the alkene under similar conditions and through a similar mechanism. It was observed that the planar configuration of intermediate B allows the H_2_ molecule to approach from the same side, leading to the syn-addition of H_2,_ resulting in the cis-product. Moreover, this study highlights that the ptC molecule CAl_3_MgH_2_^¯^, is not only an intriguing rule-breaking structure but could also catalyze hydrogenation reactions, potentially opening up new avenues for future chemistry in industrial applications. It is believed that the mechanistic insights revealed here for the hydrogenation of alkyne and alkene using the CAl_3_MgH_2_^¯^ catalyst will assist experimentalists in designing more effective main-group metal-based catalysts for hydrogenation reactions.

## Data Availability

The original contributions presented in the study are included in the article/Supplementary Material, further inquiries can be directed to the corresponding author.

## References

[B1] Aireddy D. R. Ding K. (2022). Heterolytic dissociation of H_2_ in heterogeneous catalysis. ACS Catal. 12, 4707–4723. 10.1021/acscatal.2c00584

[B2] Arrowsmith M. Hadlington T. J. Hill M. S. Kociok-Köhn G. (2012). Magnesium-catalysed hydroboration of aldehydes and ketones. Chem. Commun. 48, 4567. 10.1039/c2cc30565h 22473045

[B3] Bonyhady S. J. Jones C. Nembenna S. Stasch A. Edwards A. J. McIntyre G. J. (2010). β‐Diketiminate‐Stabilized Magnesium(I) dimers and Magnesium(II) hydride complexes: synthesis, characterization, adduct formation, and reactivity studies. Chem. - Eur. J. 16, 938–955. 10.1002/chem.200902425 19950340

[B4] Cano I. Martínez-Prieto L. M. van Leeuwen P. W. N. M. (2021). Heterolytic cleavage of dihydrogen (HCD) in metal nanoparticle catalysis. Catal. Sci. Technol. 11, 1157–1185. 10.1039/D0CY02399J

[B5] Chai J.-D. Head-Gordon M. (2008). Long-range corrected hybrid density functionals with damped atom–atom dispersion corrections. Phys. Chem. Chem. Phys. 10, 6615–6620. 10.1039/b810189b 18989472

[B6] Clark T. Chandrasekhar J. Spitznagel G. W. Schleyer P. V. R. (1983). Efficient diffuse function-augmented basis sets for anion calculations. III. The 3-21+G basis set for first-row elements, Li-F. J. Comput. Chem. 4, 294–301. 10.1002/jcc.540040303

[B7] Crabtree R. (1979). Iridium compounds in catalysis. Acc. Chem. Res. 12, 331–337. 10.1021/ar50141a005

[B8] Cui X. Burgess K. (2005). Catalytic homogeneous asymmetric hydrogenations of largely unfunctionalized alkenes. Chem. Rev. 105, 3272–3296. 10.1021/cr0500131 16159153

[B9] Das P. Pan S. Chattaraj P. K. (2023). “Planar hypercoordinate carbon,” in Atomic clusters with unusual structure, bonding and reactivity (Elsevier), 357–372. 10.1016/B978-0-12-822943-9.00021-8

[B10] de Vries J. G. Elsevier C. J. (2007). Handbook of homogeneous hydrogenation. Weinheim: Wiley VCH.

[B11] Dunne J. F. Fulton D. B. Ellern A. Sadow A. D. (2010). Concerted C−N and C−H bond Formation in a magnesium-catalyzed hydroamination. J. Am. Chem. Soc. 132, 17680–17683. 10.1021/ja108881s 21117645

[B12] Falconnet A. Magre M. Maity B. Cavallo L. Rueping M. (2019). Asymmetric magnesium‐catalyzed hydroboration by metal‐ligand cooperative catalysis. Angew. Chem. Int. Ed. 58, 17567–17571. 10.1002/anie.201908012 31642572

[B13] Fiorio J. L. Borges L. R. Neves-Garcia T. Kikuchi D. K. Guerra R. R. G. Rossi L. M. (2023). Design of gold catalysts for activation of H2 and H-donor molecules: transfer hydrogenation and CO_2_ hydrogenation. Catal. Sci. Technol. 13, 3205–3215. 10.1039/D2CY01920E

[B14] Frisch M. J. Trucks G. W. Schlegel H. B. Scuseria G. E. Robb M. A. Cheeseman J. R. (2016). *Gaussian 16*, revision B.01. Wallingford, CT: Gaussian, Inc.

[B15] Garcia L. Dinoi C. Mahon M. F. Maron L. Hill M. S. (2019a). Magnesium hydride alkene insertion and catalytic hydrosilylation. Chem. Sci. 10, 8108–8118. 10.1039/C9SC02056J 31814958 PMC6839609

[B16] Garcia L. Mahon M. F. Hill M. S. (2019b). Multimetallic alkaline-earth hydride cations. Organometallics 38, 3778–3785. 10.1021/acs.organomet.9b00493

[B17] Gonzalez C. Schlegel H. B. (1989). An improved algorithm for reaction path following. J. Chem. Phys. 90, 2154–2161. 10.1063/1.456010

[B18] Harder S. (2012). Molecular early main group metal hydrides: synthetic challenge, structures and applications. Chem. Commun. 48, 11165. 10.1039/c2cc33478j 23012695

[B19] Hill M. S. Liptrot D. J. Weetman C. (2016). Alkaline earths as main group reagents in molecular catalysis. Chem. Soc. Rev. 45, 972–988. 10.1039/C5CS00880H 26797470

[B20] Inostroza D. Leyva-Parra L. Yañez O. Solar-Encinas J. Vásquez-Espinal A. Valenzuela M. L. (2023). Searching for systems with Planar Hexacoordinate carbons. Atoms 11, 56. 10.3390/atoms11030056

[B21] Jaszczewska‐Adamczak J. A. Baczewska P. Bujok R. Mlynarski J. (2024). Magnesium‐Catalyzed asymmetric thia‐Michael addition to α,β‐Unsaturated ketones. Adv. Synth. Catal. 366, 1412–1421. 10.1002/adsc.202301414

[B22] Jin B. Yan M. Feng L. Miao C. Wang Y. (2024). CBe2H5−: unprecedented 2σ/2π double aromaticity and dynamic structural fluxionality in a planar tetracoordinate carbon cluster. Chem. - Eur. J. 30, e202304134. 10.1002/chem.202304134 38205620

[B23] Job N. Khatun M. Thirumoorthy K. Ch S. S. R. Chandrasekaran V. Anoop A. (2021). CAl_4_Mg^0/−^: global Minima with a planar tetracoordinate carbon atom. Atoms 9, 24. 10.3390/atoms9020024

[B24] Johnson N. B. Lennon I. C. Moran P. H. Ramsden J. A. (2007). Industrial-Scale synthesis and applications of asymmetric hydrogenation catalysts. Acc. Chem. Res. 40, 1291–1299. 10.1021/ar700114k 17803270

[B25] Khatun M. Roy S. Giri S. Ch S. S. R. Anoop A. Thimmakondu V. S. (2021). BAl_4_Mg^−/0/+^: global Minima with a planar tetracoordinate or hypercoordinate Boron Atom. Atoms 9, 89. 10.3390/atoms9040089

[B26] Krishnan R. Binkley J. S. Seeger R. Pople J. A. (1980). Self‐consistent molecular orbital methods. XX. A basis set for correlated wave functions. J. Chem. Phys. 72, 650–654. 10.1063/1.438955

[B27] Leyva‐Parra L. Diego L. Yañez O. Inostroza D. Barroso J. Vásquez‐Espinal A. (2021). Planar hexacoordinate carbons: half covalent, half ionic. Angew. Chem. Int. Ed. 60, 8700–8704. 10.1002/anie.202100940 33527696

[B28] Li X. Wang L.-S. Boldyrev A. I. Simons J. (1999). Tetracoordinated planar carbon in the Al_4_C^¯^ anion. A combined Photoelectron spectroscopy and *ab initio* Study. J. Am. Chem. Soc. 121, 6033–6038. 10.1021/ja9906204

[B29] Li X. Zhang H.-F. Wang L.-S. Geske G. D. Boldyrev A. I. (2000). Pentaatomic tetracoordinate planar carbon, [CAl_4_]^2−^: a new structural unit and its salt complexes. Angew. Chem. Int. Ed. 39, 3630–3632. 10.1002/1521-3773(20001016)39:20<3630::AID-ANIE3630>3.0.CO;2-R 11091420

[B30] Liang Y. Das U. K. Luo J. Diskin-Posner Y. Avram L. Milstein D. (2022). Magnesium pincer complexes and their applications in catalytic semihydrogenation of alkynes and hydrogenation of alkenes: evidence for metal–ligand cooperation. J. Am. Chem. Soc. 144, 19115–19126. 10.1021/jacs.2c08491 36194894 PMC9585592

[B31] Lindlar H. (1952). Ein neuer Katalysator für selektive Hydrierungen. Helv. Chim. Acta 35, 446–450. 10.1002/hlca.19520350205

[B32] Liptrot D. J. Hill P. M. S. Mahon M. F. (2014). Accessing the single‐electron manifold: magnesium‐mediated hydrogen release from silanes. Angew. Chem. Int. Ed. 53, 6224–6227. 10.1002/anie.201403208 24782354

[B33] Lu T. Chen F. (2012). Multiwfn: a multifunctional wavefunction analyzer. J. Comput. Chem. 33, 580–592. 10.1002/jcc.22885 22162017

[B34] Magre M. Maity B. Falconnet A. Cavallo L. Rueping M. (2019). Magnesium‐Catalyzed hydroboration of terminal and internal alkynes. Angew. Chem. Int. Ed. 58, 7025–7029. 10.1002/anie.201902188 30977970

[B35] Magre M. Szewczyk M. Rueping M. (2022). s-Block metal catalysts for the hydroboration of unsaturated bonds. Chem. Rev. 122, 8261–8312. 10.1021/acs.chemrev.1c00641 35254061 PMC9100617

[B36] Malhan A. H. Thirumoorthy K. (2024). Global minimum and a heap of low-lying isomers with planar tetracoordinate carbon in the CAl_3_MgH_2_ ^¯^ system. Phys. Chem. Chem. Phys. 26, 3804–3809. 10.1039/D3CP05841G 38240304

[B37] Malhan A. H. Sobinson S. Job N. Shajan S. Mohanty S. P. Thimmakondu V. S. (2022). Al_2_C_4_H_2_ isomers with the planar tetracoordinate carbon (ptC)/Aluminum (ptAl). Atoms 10, 112. 10.3390/atoms10040112

[B38] Meijer G. W. Kanny G. Briois J. (2022). “Natural mineral water,” in Ullmann’s encyclopedia of industrial chemistry (Wiley), 1–7. 10.1002/14356007.a04_035.pub2

[B39] Monkhorst H. J. (1968). Activation energy for interconversion of enantiomers containing an asymmetric carbon atom without breaking bonds. Chem. Commun. 1111–1112. 10.1039/c19680001111

[B40] Mukherjee D. Ellern A. Sadow A. D. (2014). Magnesium-catalyzed hydroboration of esters: evidence for a new zwitterionic mechanism. Chem. Sci. 5, 959–964. 10.1039/C3SC52793J

[B41] Nahra F. Cazin C. S. J. (2021). Sustainability in Ru- and Pd-based catalytic systems using N-heterocyclic carbenes as ligands. Chem. Soc. Rev. 50, 3094–3142. 10.1039/C8CS00836A 33475632

[B42] Naumkin F. Y. (2008). Flat-structural Motives in small alumino−carbon Clusters C_n_Al_m_ (n = 2−3, m = 2−8). J. Phys. Chem. A 112, 4660–4668. 10.1021/jp711230x 18426192

[B43] Osborn J. A. Jardine F. H. Young J. F. Wilkinson G. (1966). The preparation and properties of tris(triphenylphosphine)halogenorhodium(I) and some reactions thereof including catalytic homogeneous hydrogenation of olefins and acetylenes and their derivatives. J. Chem. Soc. 1711, 1711. 10.1039/j19660001711

[B44] Rauch M. Ruccolo S. Parkin G. (2017). Synthesis, structure, and reactivity of a terminal magnesium hydride compound with a carbatrane motif, [Tism^PriBenz^ ]MgH: a multifunctional catalyst for hydrosilylation and hydroboration. J. Am. Chem. Soc. 139, 13264–13267. 10.1021/jacs.7b06719 28901762

[B45] Reed A. E. Weinstock R. B. Weinhold F. (1985). Natural population analysis. J. Chem. Phys. 83, 735–746. 10.1063/1.449486

[B46] Revunova K. Nikonov G. I. (2015). Main group catalysed reduction of unsaturated bonds. Dalton Trans. 44, 840–866. 10.1039/C4DT02024C 25384615

[B47] Ried A. C. A. Taylor L. J. Geer A. M. Williams H. E. L. Lewis W. Blake A. J. (2019). A highly active Bidentate Magnesium Catalyst for amine‐borane dehydrocoupling: kinetic and mechanistic studies. Chem. - Eur. J. 25, 6840–6846. 10.1002/chem.201901197 30875128 PMC6563444

[B48] Roy M. M. D. Omaña A. A. Wilson A. S. S. Hill M. S. Aldridge S. Rivard E. (2021). Molecular Main Group metal hydrides. Chem. Rev. 121, 12784–12965. 10.1021/acs.chemrev.1c00278 34450005

[B49] Sarmah K. Purkayastha S. K. Guha A. K. (2023). Reversible hydrogen storage by Planar Hypercoordinate carbon clusters. Energy fuels. 37, 9598–9609. 10.1021/acs.energyfuels.3c00486

[B50] Scalmani G. Frisch M. J. (2010). Continuous surface charge polarizable continuum models of solvation. I. General formalism. J. Chem. Phys. 132, 114110. 10.1063/1.3359469 20331284

[B51] Schlegel H. B. (1982). Optimization of equilibrium geometries and transition structures. J. Comput. Chem. 3, 214–218. 10.1002/jcc.540030212

[B52] Schrock R. R. Osborn J. A. (1976). Catalytic hydrogenation using cationic rhodium complexes. II. The selective hydrogenation of alkynes to cis olefins. J. Am. Chem. Soc. 98, 2143–2147. 10.1021/ja00424a021

[B53] Sun R. Zhao X.-F. Jin B. Huo B. Bian J.-H. Guan X.-L. (2020). Influence of stepwise oxidation on the structure, stability, and properties of planar pentacoordinate carbon species CAl_5_ ^+^ . Phys. Chem. Chem. Phys. 22, 17062–17067. 10.1039/D0CP01106A 32568316

[B54] Sun R. Jin B. Huo B. Yuan C. Zhai H.-J. Wu Y.-B. (2022). Planar pentacoordinate carbon in a sulphur-surrounded boron wheel: the global minimum of CB_5_S_5_ ^+^ . Chem. Commun. 58, 2552–2555. 10.1039/D1CC07313C 35103735

[B55] Szewczyk M. Magre M. Zubar V. Rueping M. (2019). Reduction of cyclic and Linear organic carbonates using a readily available magnesium catalyst. ACS Catal. 9, 11634–11639. 10.1021/acscatal.9b04086

[B56] Thirumoorthy K. Chandrasekaran V. Cooksy A. L. Thimmakondu V. S. (2020). Kinetic stability of Si_2_C_5_H_2_ Isomer with a planar tetracoordinate carbon atom. Chem. East. 3, 13–27. 10.3390/chemistry3010002

[B57] Tomasi J. Mennucci B. Cammi R. (2005). Quantum mechanical Continuum Solvation models. Chem. Rev. 105, 2999–3094. 10.1021/cr9904009 16092826

[B58] Tsuruoka K. Koyasu K. Hirabayashi S. Ichihashi M. Tsukuda T. (2018). Size-Dependent polymorphism in aluminum Carbide cluster anions Al_n_C_2_ ^¯^: formation of acetylide-containing structures. J. Phys. Chem. C 122, 8341–8347. 10.1021/acs.jpcc.7b12767

[B59] Wang M. Kalita A. J. Orozco-Ic M. Yan G. Chen C. Yan B. (2023). Planar pentacoordinate s-block metals. Chem. Sci. 14, 8785–8791. 10.1039/D2SC05939H 37621437 PMC10445469

[B60] Wang L. Lv J. Zhang Y. Yang D. (2024). Asymmetric magnesium catalysis for important chiral scaffold synthesis. Org. Biomol. Chem. 22, 4778–4800. 10.1039/D4OB00521J 38809153

[B61] Weissermel K. Arpe H.-J. (2008). Industrial organic chemistry. John Wiley and Sons.

[B62] Wirtz L. Ghulam K. Y. Morgenstern B. Schäfer A. (2022). Constrained Geometry *ansa* ‐Half‐Sandwich Complexes of Magnesium – versatile *s* ‐block Catalysts. ChemCatChem 14, e202201007. 10.1002/cctc.202201007

[B63] Wu Y. Jiang J. Lu H. Wang Z. Perez‐Peralta N. Islas R. (2011). Starlike aluminum–carbon aromatic species. Chem. - Eur. J. 17, 714–719. 10.1002/chem.201001266 21207593

[B64] Xu J. Zhang X. Yu S. Ding Y. Bowen K. H. (2017). Identifying the hydrogenated planar tetracoordinate carbon: a combined experimental and theoretical Study of CAl_4_H and CAl_4_H^¯^ . J. Phys. Chem. Let.t 8, 2263–2267. 10.1021/acs.jpclett.7b00732 28471673

[B65] Yang L.-M. Ganz E. Chen Z. Wang Z.-X. Schleyer P. V. R. (2015). Four decades of the chemistry of Planar hypercoordinate compounds. Angew. Chem. Int. Ed. 54, 9468–9501. 10.1002/anie.201410407 26119555

[B66] Yang D. Wang L. Li D. Wang R. (2019). Magnesium catalysis in asymmetric synthesis. Chem 5, 1108–1166. 10.1016/j.chempr.2019.02.002

[B67] Yao W. Ma M. Zang S. Luo M. Zheng J. (2020). Recent advances in alkaline earth metal catalyzed hydroboration reactions. Sci. Sin. Chim. 50, 639–654. 10.1360/SSC-2020-0025

[B68] Ye D. Jiang M. Ning L. Li W.-Y. Lin L. Feng X. (2025). Chiral Magnesium(II)-Catalyzed asymmetric hydroalkylation of imine-containing vinylazaarenes through conjugate addition. Org. Lett. 27, 3601–3606. 10.1021/acs.orglett.5c00668 40156544

[B69] Zeng G. Li S. (2010). Mechanistic Insight on the hydrogenation of conjugated alkenes with H_2_ catalyzed by early main-group metal catalysts. Inorg. Chem. 49, 3361–3369. 10.1021/ic902418v 20196551

[B70] Zhang X. Emge T. J. Hultzsch K. C. (2012). A chiral phenoxyamine magnesium catalyst for the enantioselective Hydroamination/Cyclization of aminoalkenes and intermolecular hydroamination of vinyl arenes. Angew. Chem. Int. Ed. 51, 394–398. 10.1002/anie.201105079 22606691

[B71] Zhang C.-J. Wang P. Xu X.-L. Xu H.-G. Zheng W.-J. (2021). Photoelectron spectroscopy and theoretical study of Al_n_C_5_ ^−/0^ (n = 1–5) clusters: structural evolution, relative stability of star-like clusters, and planar tetracoordinate carbon structures. Phys. Chem. Chem. Phys. 23, 1967–1975. 10.1039/D0CP06081J 33470255

[B72] Zhang C. Ortíz‐Chi F. Xu X. Xu H. Merino G. Zheng W. (2023). Reconsidering the structures of C_2_Al_4_ ^−^ and C_2_Al_5_ ^−^ . Chem. - Eur. J. 29, e202301338. 10.1002/chem.202301338 37498677

[B73] Zhang M. Gu J. Zhao Y. (2025). Au^I^ complexes with planar tetracoordinate carbon and their catalytic activity for the rearrangement of allylic acetates: a computational Study. ACS Omega 10, 12514–12521. 10.1021/acsomega.5c00089 40191324 PMC11966577

[B74] Zhao X.-F. Bian J.-H. Huang F. Yuan C. Wang Q. Liu P. (2018). Stabilization of beryllium-containing planar pentacoordinate carbon species through attaching hydrogen atoms. RSC Adv. 8, 36521–36526. 10.1039/C8RA07664B 35558954 PMC9088823

[B75] Zhou Q. (2016). Transition‐Metal catalysis and organocatalysis: where can progress be expected? Angew. Chem. Int. Ed. 55, 5352–5353. 10.1002/anie.201509164 26662619

