# Supplementary material for: Catalytic hydrogenation of alkyne with planar tetracoordinate carbon in CAl3MgH2 ¯ system
Source: Front Chem. 2025 Oct 31;13:1672968. doi: 10.3389/fchem.2025.1672968 (PMC12616281; doi:10.3389/fchem.2025.1672968)
Supplement: Supplementary file 3 [file DataSheet1.docx]

**Catalytic Hydrogenation of Alkyne with Planar Tetracoordinate Carbon in CAl_3_MgH_2_^¯^ System**

Abdul Hamid Malhan^1^, Krishnan Thirumoorthy^1,2^*

^1^Department of Chemistry, School of Advanced Sciences, Vellore Institute of Technology, Vellore 632 014, Tamil Nadu, India

^2^School of Computer Science and Engineering, Vellore Institute of Technology, Vellore 632 014, Tamil Nadu, India

***Correspondence:**

Krishnan Thirumoorthy

[thirumoorthy.krishnan@vit.ac.in](mailto:thirumoorthy.krishnan@vit.ac.in) & kthirumoorthy@gmail.com


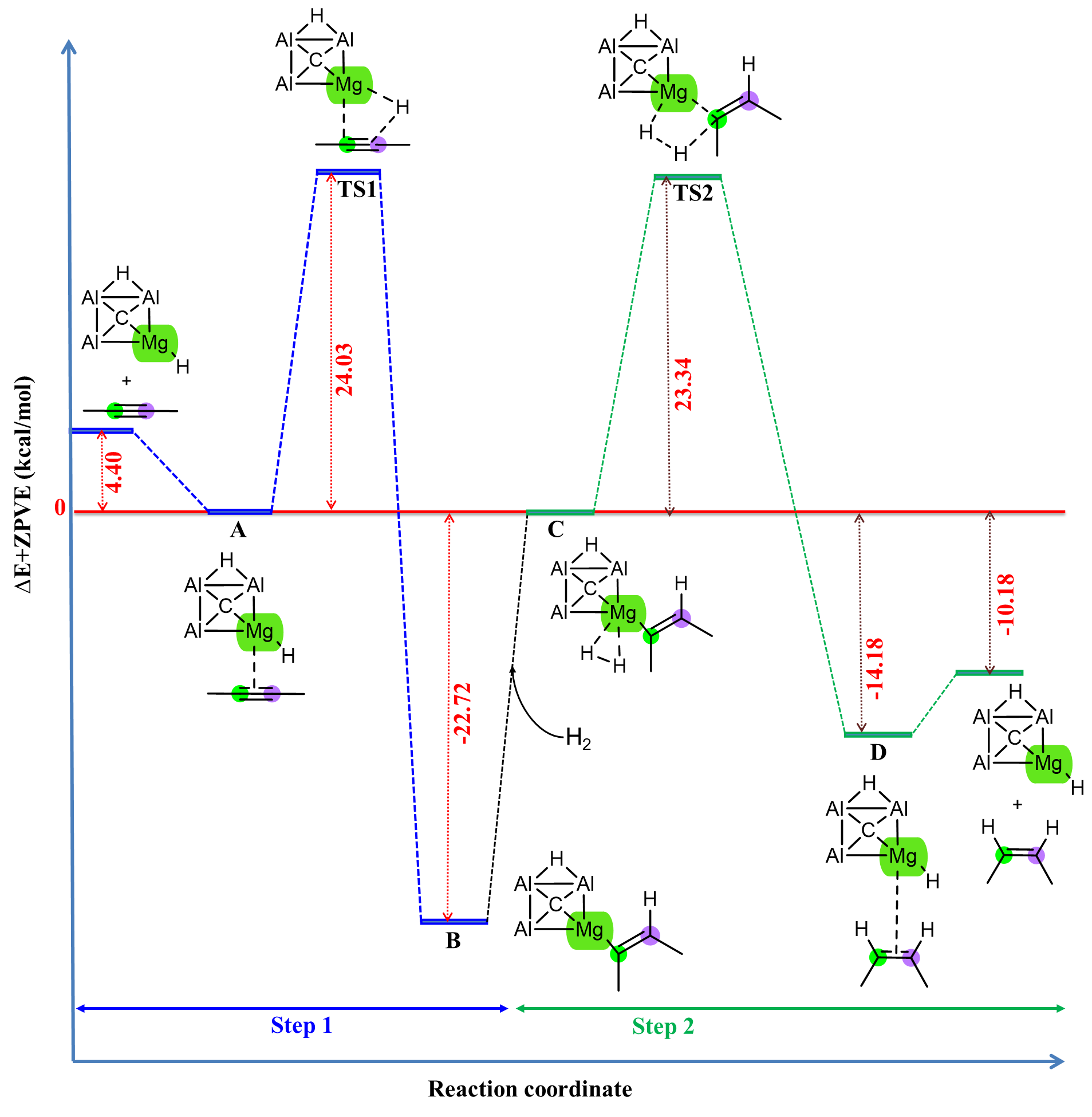


**Figure S1.** Zero-point corrected energy profile in kcal/mol of hydrogenation of 2-butyne in the PCM solvation of toluene using CAl_3_MgH_2_^¯^ catalyst. The reaction proceeds via two transition states with activation barriers of 24.03 kcal/mol for **TS1** and 23.34 kcal/mol for **TS2**. All energies are calculated at ωB97XD/6-311++G(2d,2p) level of theory.

**
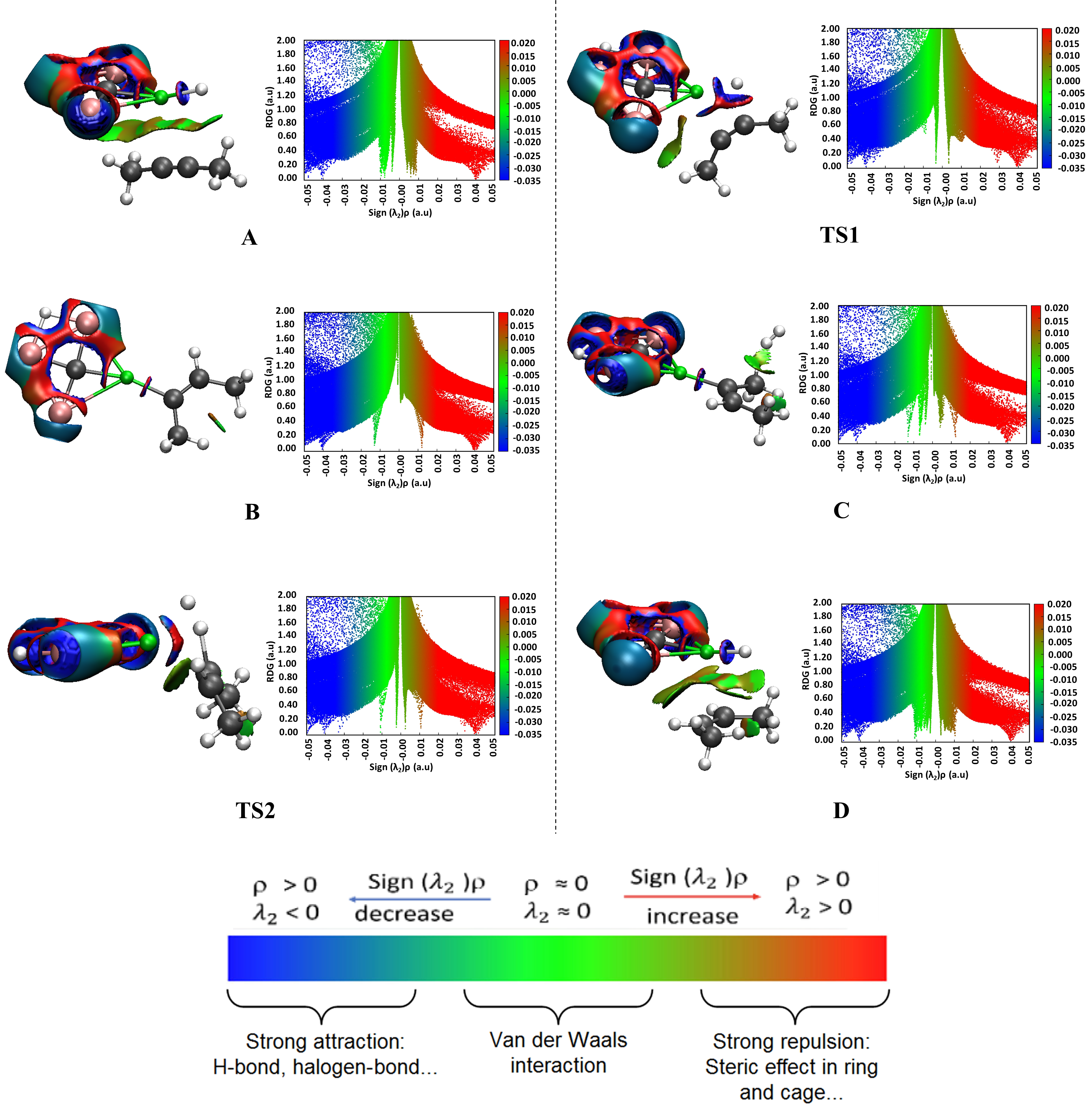
**

**Figure S2.** Non-covalent interaction, 3D isosurfaces (on left) and 2D reduced density gradient graphs (on right) with complete interaction for optimized geometries of all the stationary points involved in the reaction pathway of hydrogenation of 2-butyne in PCM solvation of toluene using CAl_3_MgH_2_^¯^ catalyst at ωB97XD/6-311++G(2d,2p) level of theory. The reaction initiates with van der Waals interactions (green isosurface) that bring the reactants together in the initial complex. As the system approaches the transition state, these evolve into strong, electrostatic interactions (blue isosurface), which are critical for product formation. Isosurfaces are colored as: strong attraction (blue), van der Waals interaction (green), and repulsive interaction (red).


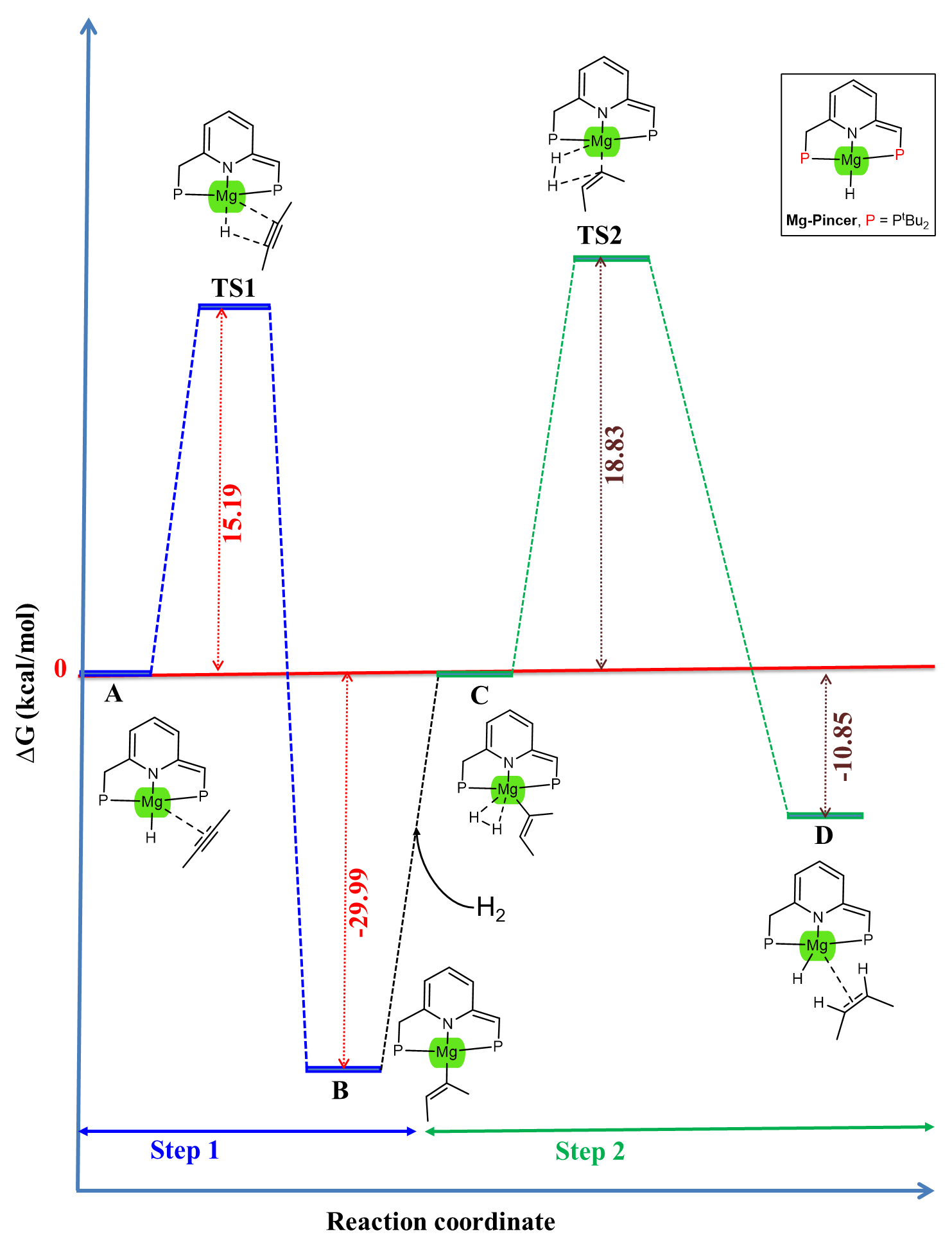


**Figure S3.** Gibbs free energy profile in kcal/mol for hydrogenation of 2-butyne in PCM solvation of toluene using magnesium pincer catalyst. The reaction proceeds via two transition states with activation barriers of 15.19 kcal/mol for **TS1** and 18.83 kcal/mol for **TS2**. All energies are calculated at ωB97XD/6-311++G(2d,2p) level of theory.


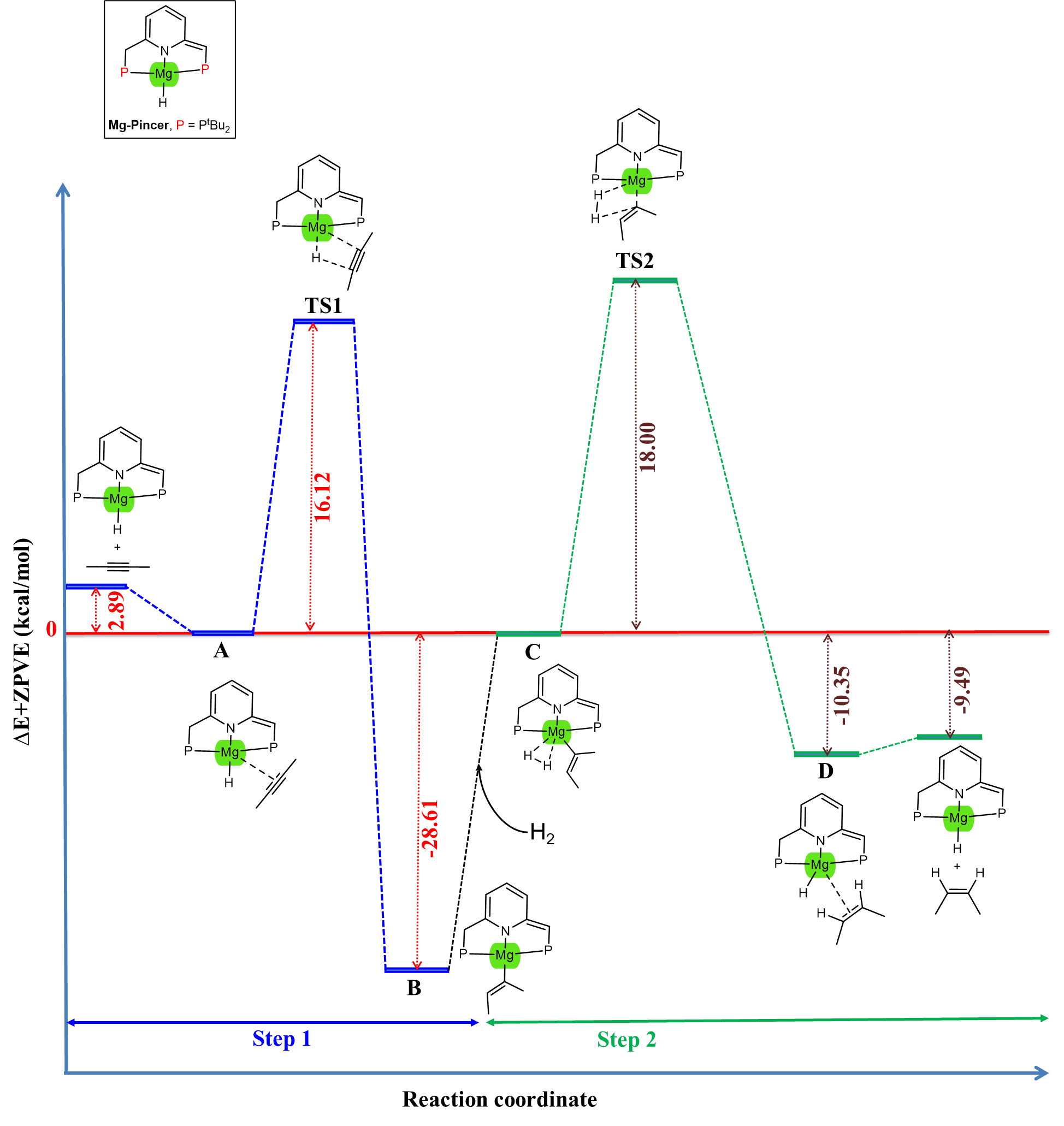


**Figure S4.** Zero-point corrected energy profile in kcal/mol of hydrogenation of 2-butyne in the PCM solvation of toluene using magnesium pincer catalyst. The reaction proceeds via two transition states with activation barriers of 16.12 kcal/mol for **TS1** and 18.00 kcal/mol for **TS2**. All energies are calculated at ωB97XD/6-311++G(2d,2p) level of theory.


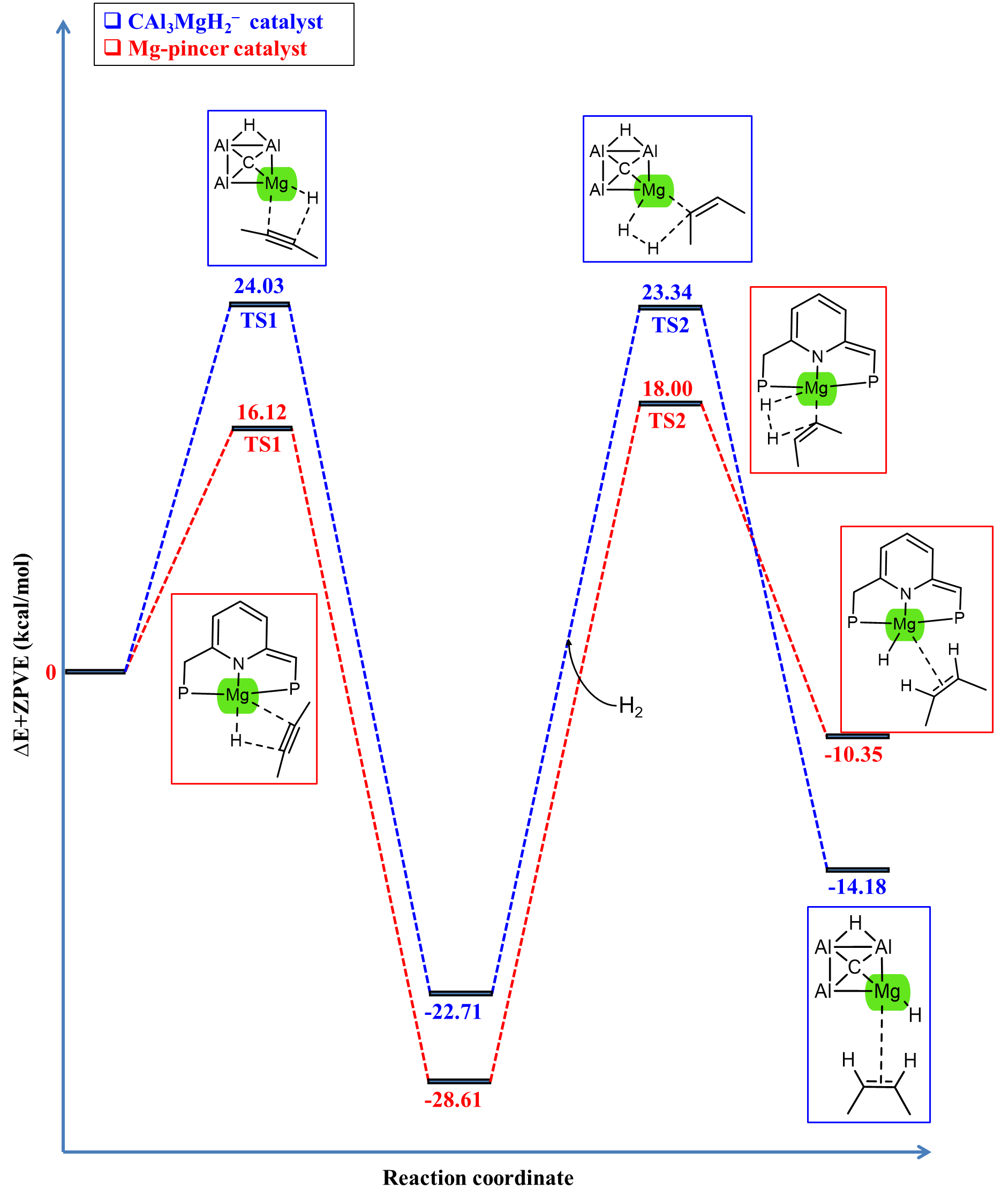


**Figure S5.** Comparison of zero-point corrected energy profile in kcal/mol for hydrogenation of 2-butyne in PCM solvation of toluene between CAl_3_MgH_2_^¯^ and magnesium pincer catalyst at ωB97XD/6-311++G(2d,2p) level of theory.

**
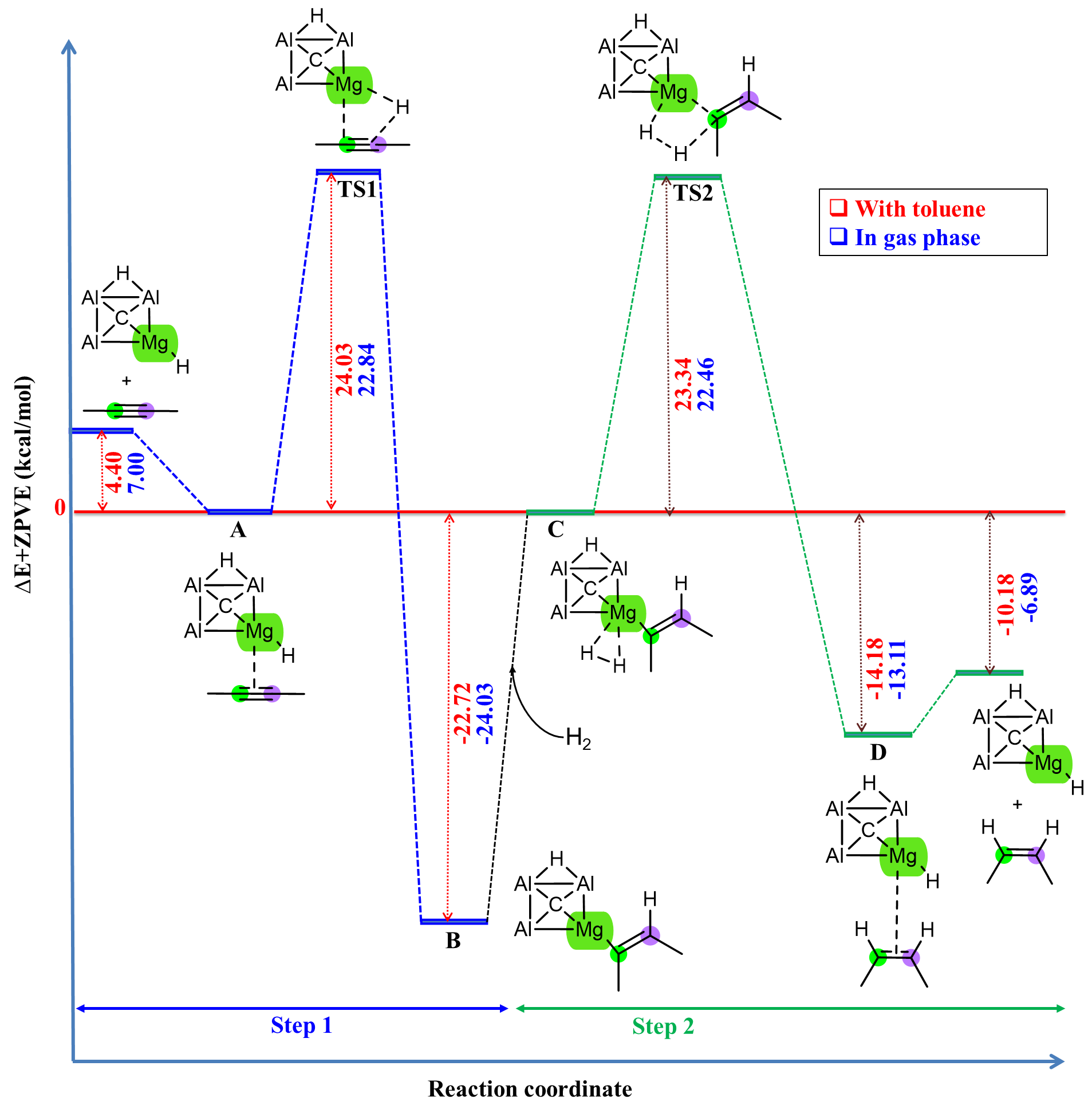
**

**Figure S6.** Comparison of zero-point corrected energy profile in kcal/mol for hydrogenation of 2-butyne using CAl_3_MgH_2_^¯^ in PCM solvation of toluene and gas phase. The lower activation barrier in the gas phase favors the reaction kinetically compared to the solvent phase. All energies are calculated at ωB97XD/6-311++G(2d,2p) level of theory.


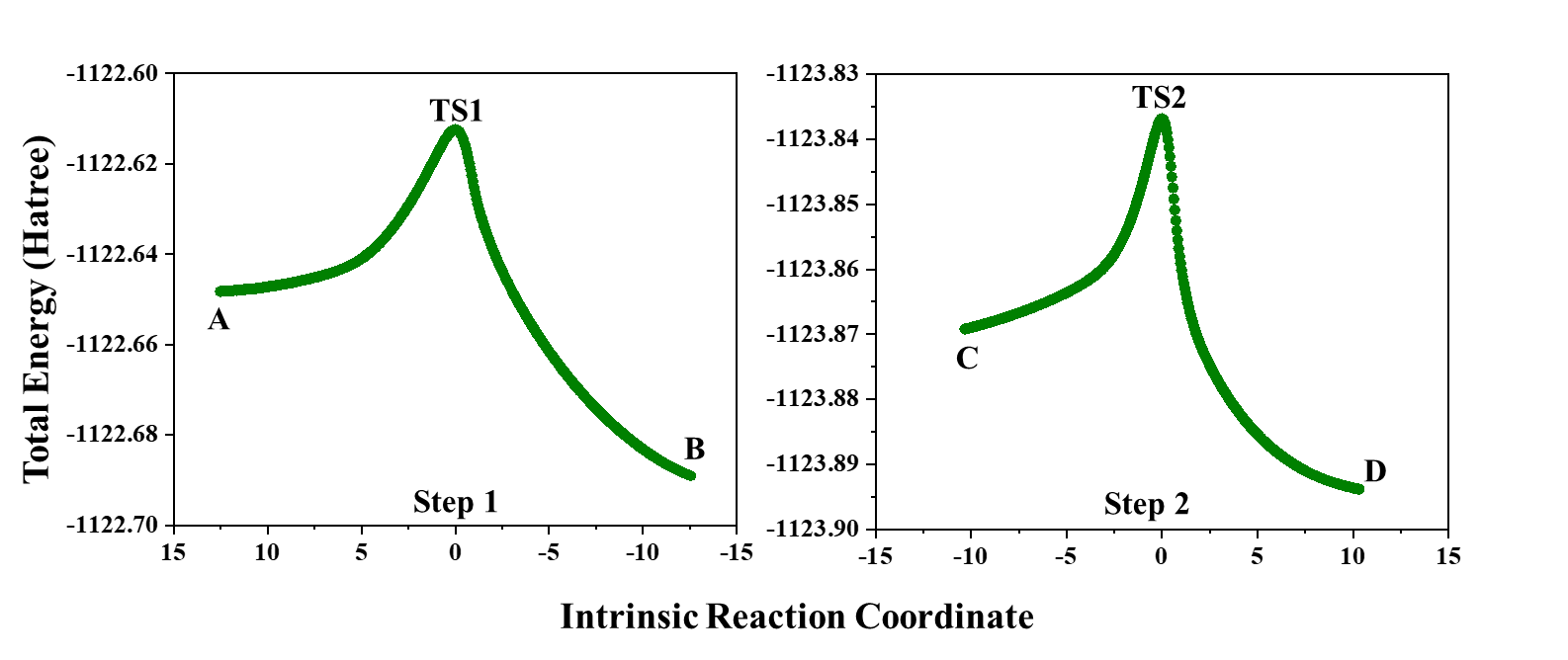


**Figure S7.** Intrinsic reaction coordinate pathway for hydrogenation of 2-butyne in the gas phase using CAl_3_MgH_2_^¯^ catalyst. Both transition states are truly connected to their adjacent local minima. Intrinsic reaction coordinate analysis is performed at ωB97XD/6-311++G(2d,2p) level of theory.

**
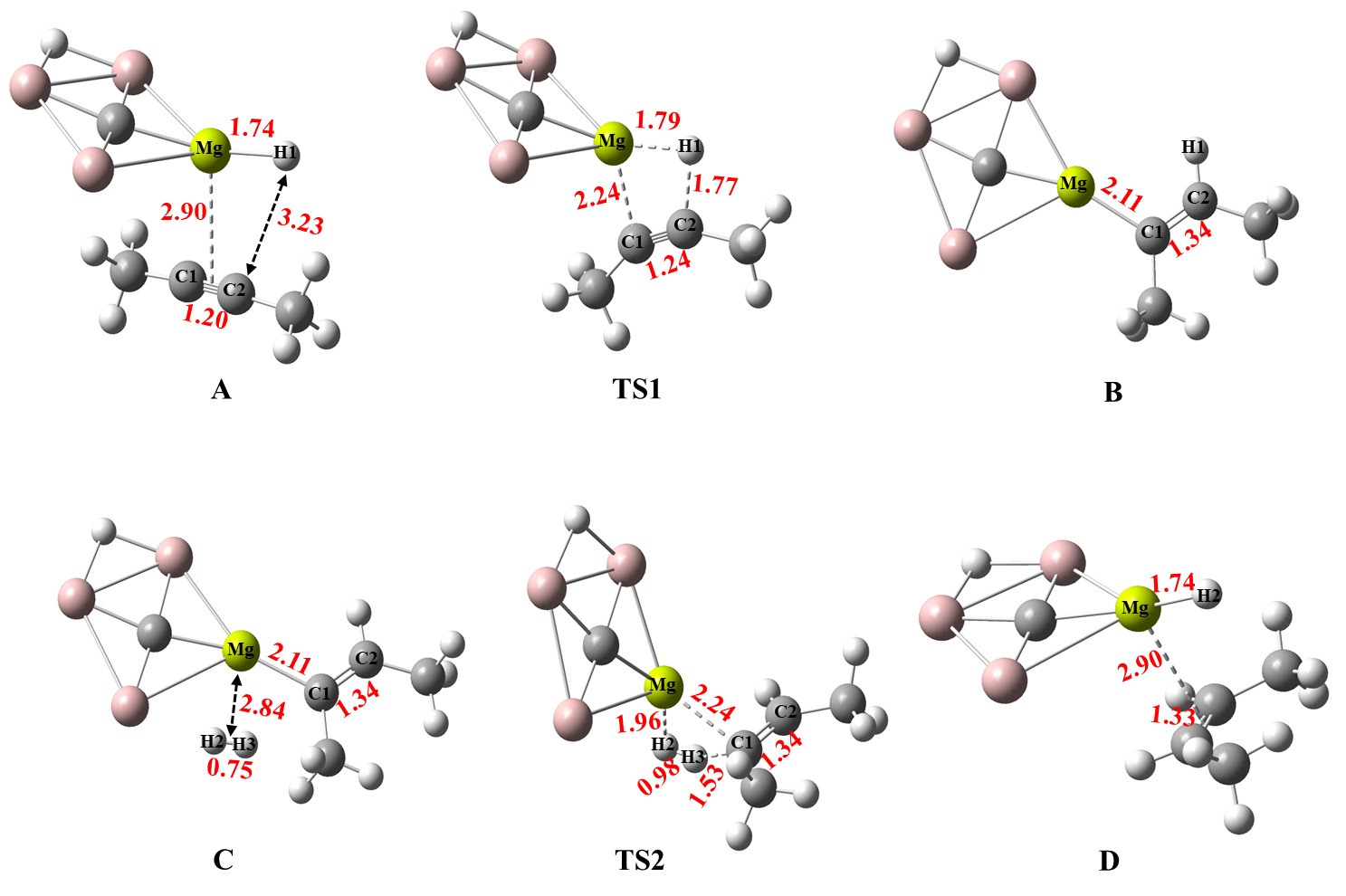
**

**Figure S8.** Calculated bond lengths in Å for optimized structures of all the stationary points involved in the reaction pathway of hydrogenation of 2-butyne in the gas phase using CAl_3_MgH_2_^¯^ catalyst at ωB97XD/6-311++G(2d,2p) level of theory. The elongation of the Mg–H1 bond in **TS1** confirms the transfer of H1. In **TS2**, the elongation of H_2_ confirms its cleavage.

**
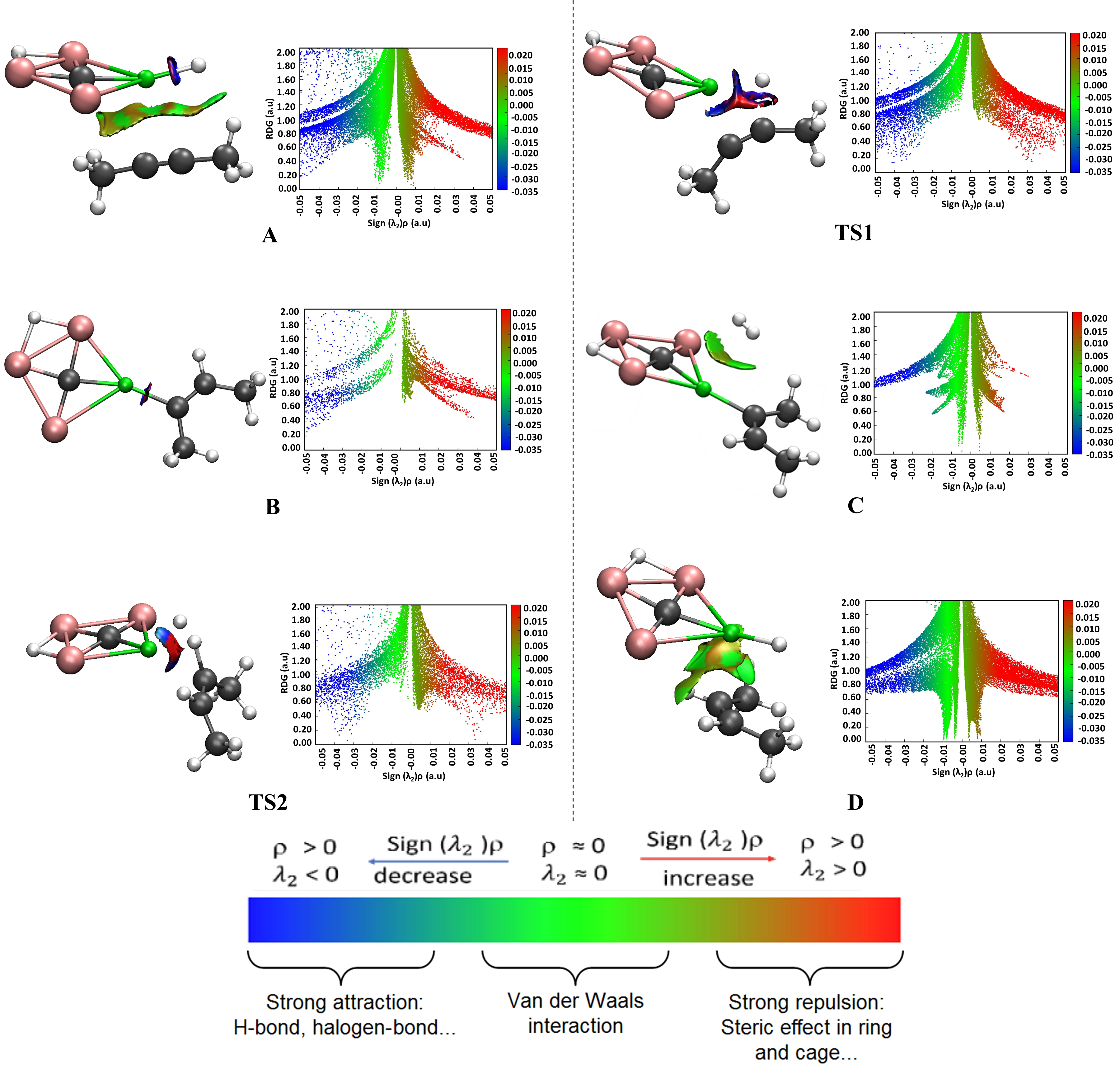
**

**Figure S9.** Non-covalent interaction, 3D isosurfaces (on left) and 2D reduced density gradient graphs (on right) for the specific interactions associated with bond breaking and formation involved in the reaction pathway of hydrogenation of 2-butyne in the gas phase using CAl_3_MgH_2_^¯^ catalyst at ωB97XD/6-311++G(2d,2p) level of theory. The reaction initiates with van der Waals interactions (green isosurfaces) that bring the reactants together in the initial complex. As the system approaches the transition state, these evolve into strong, electrostatic interactions (blue isosurface), which are critical for product formation. Isosurfaces are colored as: strong attraction (blue), van der Waals interaction (green), and repulsive interaction (red).


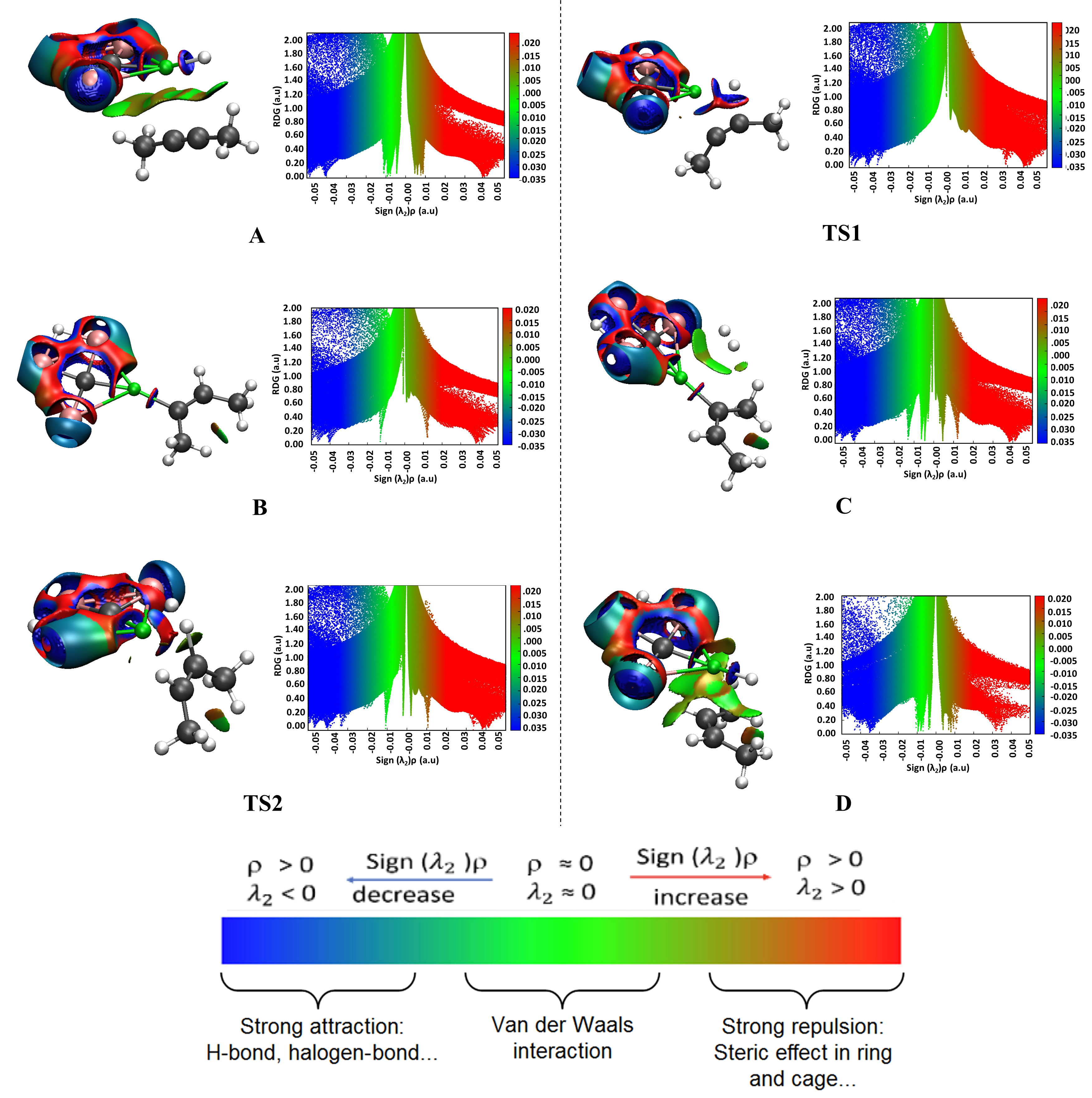


**Figure S10.** Non-covalent interaction, 3D isosurfaces (on left) and 2D reduced density gradient graphs (on right) with complete interaction for optimized geometries of all the stationary points involved in the reaction pathway of hydrogenation of 2-butyne in the gas phase using CAl_3_MgH_2_^¯^ catalyst at ωB97XD/6-311++G(2d,2p) level of theory. The reaction initiates with van der Waals interactions (green isosurfaces) that bring the reactants together in the initial complex. As the system approaches the transition state, these evolve into strong, electrostatic interactions (blue isosurface), which are critical for product formation. Isosurfaces are colored as: strong attraction (blue), van der Waals interaction (green), and repulsive interaction (red).

**
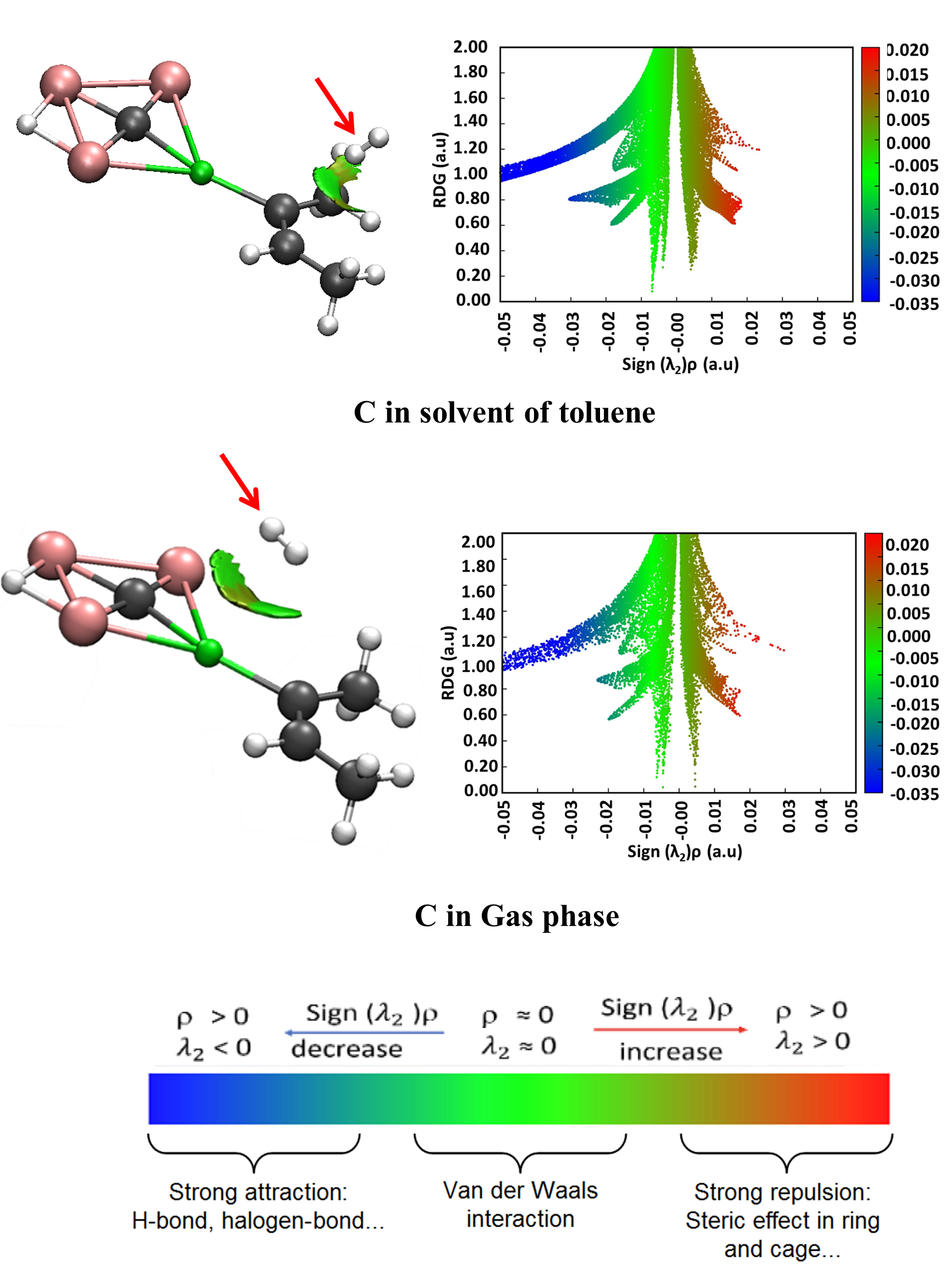
**

**Figure S11.** Non-covalent interaction, 3D isosurfaces (on left) and 2D reduced density gradient graphs (on right) for the specific interactions associated with H_2_ in the reactant state **C** with PCM solvation of toluene and gas phase at ωB97XD/6-311++G(2d,2p) level of theory. In the solvent phase, H_2_ interacts with the C=C bond, whereas in the gas phase, H_2_ interacts with the Mg atom. Isosurfaces are colored as: strong attraction (blue), van der Waals interaction (green), and repulsive interaction (red).


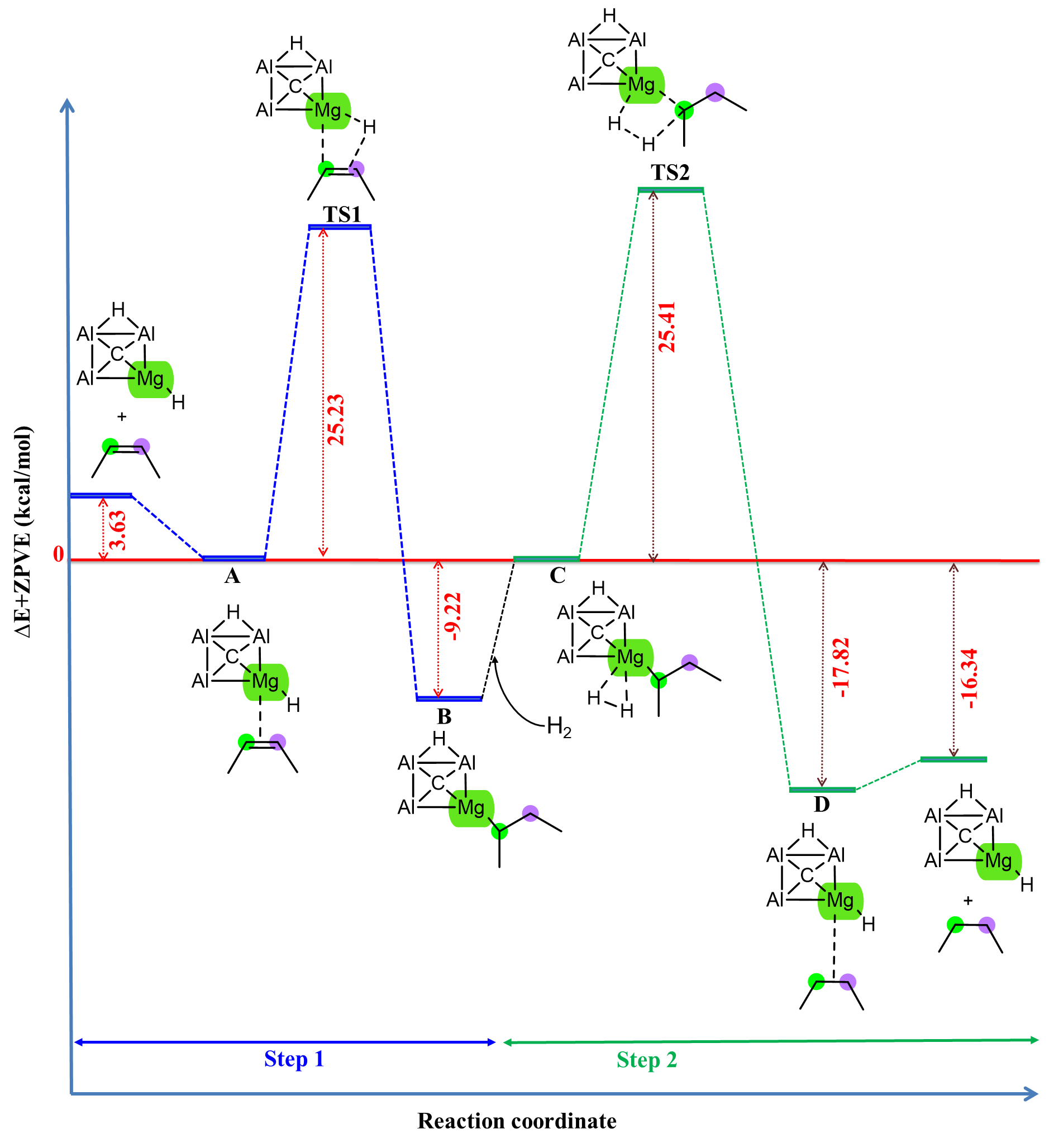


**Figure S12.** Zero-point corrected energy profile in kcal/mol of hydrogenation of 2-butene in the solvent phase of toluene using CAl_3_MgH_2_^¯^ catalyst. The reaction proceeds via two transition states with activation barriers of 25.23 kcal/mol for **TS1** and 25.41 kcal/mol for **TS2**. All energies are calculated at ωB97XD/6-311++G(2d,2p) level of theory.

**
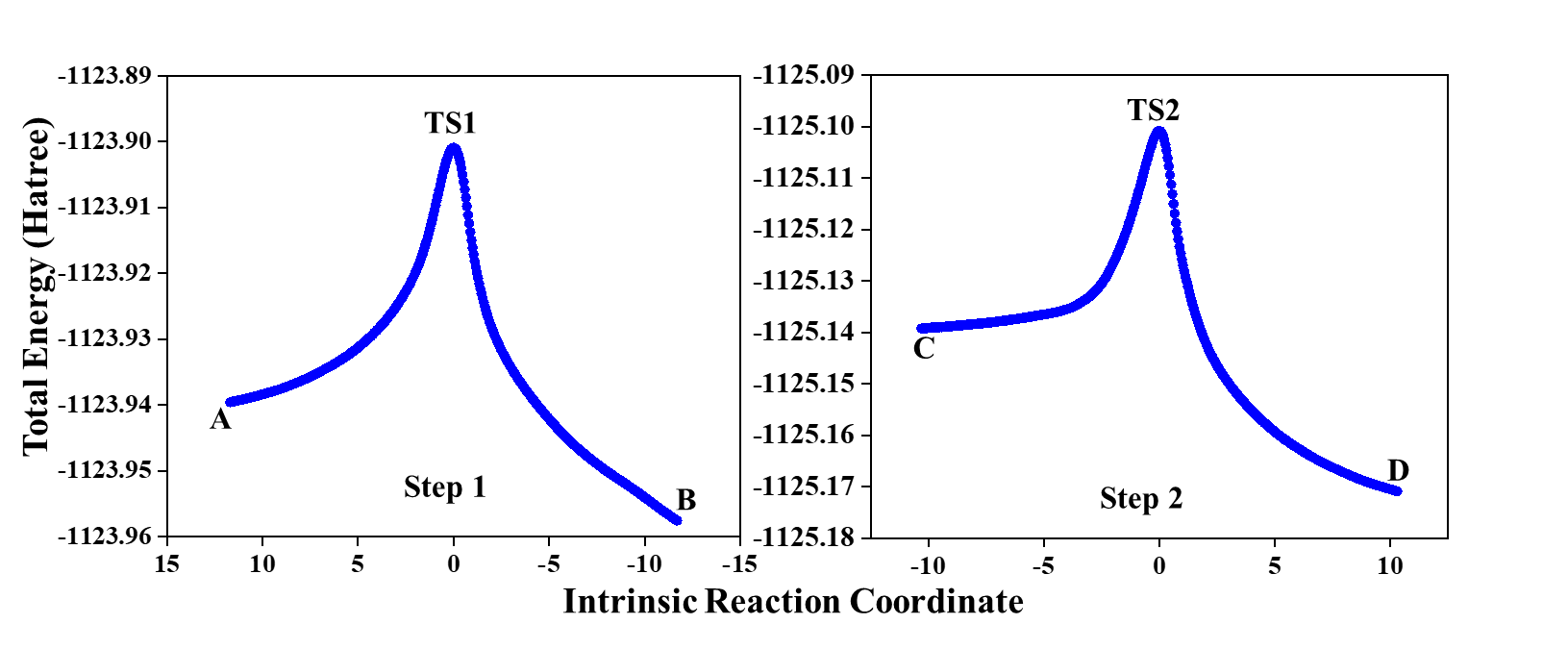
**

**Figure S13.** Intrinsic reaction coordinate pathway for hydrogenation of 2-butene in PCM solvation of toluene using CAl_3_MgH_2_^¯^ catalyst. Both transition states are truly connected to their adjacent local minima. Intrinsic reaction coordinate analysis is performed at ωB97XD/6-311++G(2d,2p) level of theory.

**
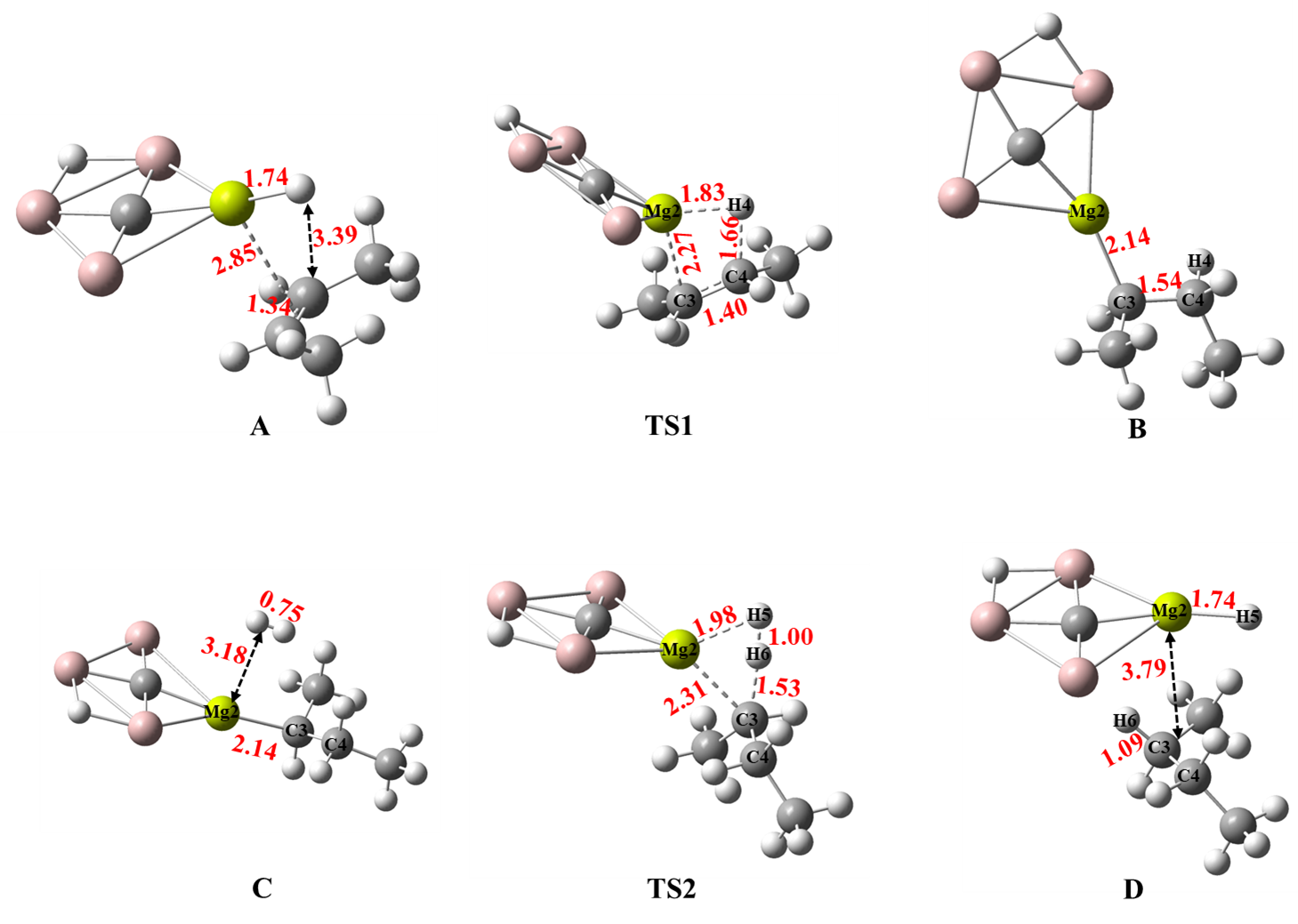
**

**Figure S14.** Calculated bond lengths in Å for optimized structures of all the stationary points involved in the reaction pathway of hydrogenation of 2-butene in PCM solvation of toluene using CAl_3_MgH_2_^¯^ catalyst. The elongation of the Mg–H1 bond in **TS1** confirms the transfer of H1. In **TS2**, the elongation of H_2_ confirms its cleavage.

**Table S1.** Total energy (in a.u)**,** zero-point vibrational energy (ZPVE; in a.u), ZPVE corrected total energy (E+ZPVE; in a.u), relative energy (ΔE + ZPVE; in kcal/mol), and the number of imaginary frequencies (NImag) for optimized structures of the stationary points involved in the reaction pathway for hydrogenation of 2-butyne in PCM solvation of toluene using CAl_3_MgH_2_^¯^ catalyst at ωB97XD/6-311++G(2d,2p) level of theory. Their Zero-point corrected energy profile is given in **Figure S1**.

|  | Energy  (a.u) | ZPVE  (a.u) | E+ZPVE  (a.u) | ΔE+ZPVE  (kcal/mol) | NImag |
| --- | --- | --- | --- | --- | --- |
| A | -1122.6928 | 0.10495 | -1122.5879 | 0 | 0 |
| TS1 | -1122.6547 | 0.105103 | -1122.5496 | 24.03 | 1 |
| B | -1122.7353 | 0.111223 | -1122.6241 | -22.72 | 0 |
| C | -1123.9141 | 0.123343 | -1123.7907 | 0 | 0 |
| TS2 | -1123.8778 | 0.12426 | -1123.7535 | 23.34 | 1 |
| D | -1123.9424 | 0.129113 | -1123.8133 | -14.18 | 0 |

**Table S2.** Decomposition analysis of Wiberg bond order in natural atomic orbital basis for the transition states in the reaction pathway of hydrogenation of 2-butyne in PCM solvation of toluene using CAl_3_MgH_2_^¯^ catalyst calculated at ωB97XD/6-311++G(2d,2p) level of theory. (Labels are followed as given in **Figure 5**)

|  | **Natural atomic orbital type** | **Contribution** |
| --- | --- | --- |
| **TS1** | 3s(Mg)-1s(H1)  2p_y_(C2)-1s(H1)  C1-Mg | 0.10  0.11  - |
| **TS2** | 2p_z_(C1)-1s(H3)  Mg-H2  C1-Mg | 0.19  -  - |

**Table S3.** Total energy (in a.u)**,** zero-point vibrational energy (ZPVE; in a.u), ZPVE corrected total energy (E+ZPVE; in a.u), relative energy (ΔE + ZPVE; in kcal/mol), and the number of imaginary frequencies (NImag) for optimized structures of the stationary points involved in the reaction pathway for hydrogenation of 2-butyne in PCM solvation of toluene using magnesium pincer catalyst at ωB97XD/6-311++G(2d,2p) level of theory. Their Zero-point corrected energy profile is given in **Figure S4**.

|  | Energy  (a.u) | ZPVE  (a.u) | E+ZPVE  (a.u) | ΔE+ZPVE  (kcal/mol) | NImag |
| --- | --- | --- | --- | --- | --- |
| A | -1995.9519 | 0.705252 | -1995.2467 | 0 | 0 |
| TS1 | -1995.9241 | 0.703081 | -1995.221 | 16.12 | 1 |
| B | -1996.001 | 0.708653 | -1995.2923 | -28.61 | 0 |
| C | -1997.1803 | 0.727294 | -1996.453 | 0 | 0 |
| TS2 | -1997.1478 | 0.723554 | -1996.4243 | 18.00 | 1 |
| D | -1997.1969 | 0.727377 | -1996.4695 | -10.35 | 0 |

**Table S4.** Comparison of Gibbs free energy profile and their energy differences in kcal/mol for hydrogenation of 2-butyne in the PCM solvation of toluene between CAl_3_MgH_2_^¯^ and magnesium pincer catalyst at ωB97XD/6-311++G(2d,2p) level of theory. The Gibbs free energy profile using CAl_3_MgH_2_^¯^ and magnesium pincer catalyst is given in **Figure 1** and **Figure S3**, respectively.

| Gibbs free energy (kcal/mol) | | | |
| --- | --- | --- | --- |
|  | **CAl_3_MgH_2_^¯^ catalyst** | **Magnesium pincer catalyst** | **ΔG** |
| **A** | 0.00 | 0.00 | 0.00 |
| **TS1** | 25.67 | 15.19 | 10.48 |
| **B** | -21.46 | -29.99 | 8.53 |
| **C** | 0.00 | 0.00 | 0.00 |
| **TS2** | 25.04 | 18.83 | 6.21 |
| **D** | -11.98 | -10.85 | 1.13 |

**Table S5.** Natural atomic charges (|e|) on atoms involved in the reaction pathway of hydrogenation of 2-butyne in the gas phase using CAl_3_MgH_2_^¯^ catalyst calculated at ωB97XD/6-311++G(2d,2p) level of theory. (Labels are followed as given in **Figure S8**).

|  | **Mg** | **C1** | **C2** | **H1** | **H2** | **H3** |
| --- | --- | --- | --- | --- | --- | --- |
| **A** | 1.57 | -0.04 | -0.06 | -0.72 | - | - |
| **TS1** | 1.68 | -0.40 | -0.06 | -0.47 | - | - |
| **B** | 1.64 | -0.61 | -0.27 | 0.17 | - | - |
| **TS2** | 1.70 | -0.56 | -0.23 | 0.17 | -0.35 | 0.10 |
| **D** | 1.54 | -0.21 | -0.23 | 0.17 | -0.72 | 0.20 |

**Table S6.** Natural atomic charges (|e|) on atoms involved in the reaction pathway of hydrogenation of 2-butene in PCM solvation of toluene using CAl_3_MgH_2_^¯^ catalyst calculated at ωB97XD/6-311++G(2d,2p) level of theory. (Labels are followed as given in **Figure S14**)

|  | **Mg2** | **C3** | **C4** | **H4** | **H5** | **H6** |
| --- | --- | --- | --- | --- | --- | --- |
| **A** | 1.59 | -0.23 | -0.23 | -0.74 | - | - |
| **TS1** | 1.72 | -0.69 | -0.22 | -0.40 | - | - |
| **B** | 1.67 | -0.84 | -0.40 | 0.18 | - | - |
| **TS2** | 1.75 | -0.79 | -0.40 | 0.18 | -0.34 | 0.07 |
| **D** | 1.59 | -0.40 | -0.39 | - | -0.73 | 0.22 |

**Table S7.** Cartesian coordinates of stationary points of hydrogenation of 2-butyne in PCM solvation of toluene using CAl_3_MgH_2_^¯^ catalyst at ωB97XD/6-311++G(2d,2p) level of theory.

| **A** | **TS1** |
| --- | --- |
| C -2.965483 -0.135088 -0.275376  C -2.128710 -0.784568 -0.847278  Mg -0.583908 0.449499 1.267580  C 1.271570 0.314221 0.241344  Al 1.196036 1.931176 -0.777591  Al 1.530149 -1.395434 1.164562  Al 3.094171 0.027920 -0.483140  H -1.712824 1.145600 2.410711  H 3.164025 -1.518829 0.425641  C -4.039765 0.628468 0.356347  C -1.190948 -1.593193 -1.623595  H -4.928235 0.007630 0.471245  H -3.724373 0.980190 1.338780  H -4.298664 1.494769 -0.252333  H -1.642410 -1.874839 -2.575131  H -0.273542 -1.035819 -1.807825  H -0.921684 -2.499342 -1.080506 | C 2.868369 -0.309689 -0.229830  C 2.225785 0.392172 0.570715  Mg 0.529527 -0.748197 -0.325274  C -1.459526 -0.032155 -0.206049  Al -1.195193 1.766886 -0.787547  Al -1.873056 -1.708789 0.727820  Al -3.414971 0.286670 -0.188874  H 1.931844 -1.457428 -1.201821  H -3.615840 -1.290791 0.666359  C 4.125201 -0.741390 -0.876664  C 2.198746 1.455367 1.601098  H 4.952502 -0.166038 -0.453315  H 4.304961 -1.801972 -0.707964  H 4.085965 -0.572376 -1.951591  H 3.181755 1.899261 1.774834  H 1.506293 2.246067 1.308212  H 1.828610 1.053847 2.544774 |
| **B** | **C** |
| C 3.241729 -0.788585 -0.000304  C 2.416031 0.270354 0.000046  Mg 0.335284 -0.123764 -0.000001  C -1.774706 0.053516 0.000055  Al -1.969596 1.955132 0.000085  Al -1.769644 -1.909994 0.000040  Al -3.754990 -0.108114 -0.000129  H 2.802268 -1.787132 -0.000641  H -3.553837 -1.906552 -0.000068  C 4.751360 -0.833595 -0.000311  C 2.949517 1.688674 0.000493  H 5.213366 0.151749 0.000097  H 5.119733 -1.374066 0.876045  H 5.119769 -1.373365 -0.877086  H 4.039901 1.778584 0.000447  H 2.578340 2.236522 -0.870395  H 2.578454 2.235939 0.871796 | C 3.149974 -0.826921 -0.100171  C 2.330084 0.235789 -0.152347  Mg 0.247759 -0.139868 -0.060370  C -1.859104 0.054842 0.004862  Al -2.039953 1.957998 0.013950  Al -1.869687 -1.908472 0.020626  Al -3.840571 -0.090914 0.026914  H 2.705559 -1.818717 -0.006249  H -3.654079 -1.890331 0.043240  C 4.658069 -0.881717 -0.144516  C 2.870554 1.645507 -0.279010  H 5.121667 0.093326 -0.281009  H 5.053452 -1.309632 0.781396  H 4.995777 -1.529427 -0.957896  H 3.958536 1.724750 -0.360929  H 2.440872 2.143214 -1.152880  H 2.566150 2.247709 0.582355  H 3.662798 0.467699 2.353918  H 4.031441 0.722864 2.950213 |
| **TS2** | **D** |
| C 3.120929 -0.652433 0.194384  C 2.354472 0.436046 0.327436  Mg 0.294906 -0.293305 0.815033  C -1.665683 0.027525 0.108342  Al -1.750818 1.939612 0.105042  Al -1.734762 -1.929668 -0.065364  Al -3.551902 -0.013772 -0.503687  H 2.912373 -1.489976 0.870796  H -3.427579 -1.816263 -0.607282  C 4.271702 -0.909534 -0.736023  C 2.557164 1.708976 -0.452191  H 4.474716 -0.066730 -1.394396  H 5.181502 -1.132456 -0.172670  H 4.066634 -1.782166 -1.360372  H 3.520477 1.775031 -0.966658  H 1.772228 1.834328 -1.203293  H 2.475090 2.573511 0.210203  H 1.798528 0.215717 1.726020  H 1.342939 -0.205056 2.497685 | C -2.088197 0.627961 -0.850141  C -2.361184 -0.677884 -0.781848  Mg -0.381942 -0.284240 1.235361  C 1.463310 0.048108 0.239083  Al 1.511348 1.959076 0.247536  Al 1.538892 -1.894794 0.002158  Al 3.264964 0.068585 -0.582391  H -1.602840 -0.210029 2.487908  H 3.172943 -1.726031 -0.711871  H -1.714235 -1.345122 -1.343564  H -1.239451 0.919752 -1.460674  C -3.481594 -1.342278 -0.043115  C -2.828956 1.753546 -0.195480  H -4.173489 -1.800992 -0.753546  H -3.099226 -2.142813 0.592304  H -4.040331 -0.653884 0.586180  H -3.353308 2.344865 -0.949952  H -3.553545 1.411742 0.540053  H -2.131152 2.429411 0.302909 |

**Table S8.** Cartesian coordinates of stationary points of hydrogenation of 2-butyne in PCM solvation of toluene using magnesium pincer catalyst at ωB97XD/6-311++G(2d,2p) level of theory.

| **A** | **TS1** |
| --- | --- |
| P 2.644554 -0.027655 0.227289  P -2.597271 0.166892 0.229062  Mg 0.077496 0.553381 0.482306  N -0.062524 -1.146083 -0.909016  C 2.316805 -1.569964 -0.558278  H 3.105466 -2.304736 -0.661083  C 1.063480 -1.956243 -1.000112  C 0.909358 -3.271059 -1.578168  H 1.777816 -3.914609 -1.604639  C -0.278607 -3.684803 -2.072330  H -0.377049 -4.673656 -2.502372  C -1.394084 -2.816569 -2.026510  H -2.351755 -3.107860 -2.430793  C -1.231471 -1.587860 -1.446662  C -2.412965 -0.649728 -1.410140  H -3.314508 -1.166816 -1.733812  H -2.231772 0.158300 -2.118317  C 3.979895 0.795571 -0.831636  C 5.065137 -0.176394 -1.306186  H 5.647839 -0.591106 -0.487456  H 5.755562 0.353561 -1.968439  H 4.631069 -1.001056 -1.870858  C 4.620931 1.985120 -0.112909  H 3.869152 2.662695 0.296084  H 5.229705 2.551934 -0.822372  H 5.275727 1.669554 0.698151  C 3.241422 1.317097 -2.070966  H 2.685923 0.517185 -2.563100  H 3.964741 1.716495 -2.786818  H 2.547879 2.114471 -1.808032  C 3.329609 -0.438890 1.943637  C 4.714137 -1.089302 1.932811  H 4.746293 -1.960161 1.276318  H 4.965027 -1.425897 2.942642  H 5.491343 -0.392290 1.621745  C 3.344651 0.825736 2.813686  H 4.057646 1.568288 2.460993  H 3.630507 0.557506 3.834766  H 2.353140 1.279233 2.849865  C 2.332526 -1.425615 2.570618  H 1.333374 -0.992414 2.641224  H 2.658336 -1.659658 3.587597  H 2.272673 -2.353311 2.003319  C -3.160792 -1.220875 1.382329  C -1.893952 -1.991986 1.798809  H -1.398358 -2.467927 0.954678  H -2.179496 -2.778635 2.501533  H -1.180063 -1.340657 2.305785  C -4.140352 -2.210361 0.744125  H -5.056725 -1.735957 0.399978  H -4.416459 -2.964284 1.485751  H -3.684305 -2.735050 -0.095379  C -3.767146 -0.620494 2.656555  H -3.100629 0.115600 3.108262  H -3.918846 -1.420634 3.384626  H -4.734867 -0.154359 2.479812  C -3.969683 1.430600 -0.045207  C -5.368955 0.838418 -0.228844  H -5.397578 0.116323 -1.045639  H -6.068774 1.641394 -0.474397  H -5.735706 0.352123 0.672797  C -3.618303 2.234925 -1.307244  H -2.596909 2.614612 -1.276376  H -4.289221 3.094353 -1.370934  H -3.753417 1.651615 -2.217565  C -3.955627 2.408264 1.139944  H -4.212328 1.930952 2.082327  H -4.684544 3.202327 0.960741  H -2.973752 2.868714 1.254081  H 0.035119 1.101754 2.168568  C -0.001979 2.437087 -1.493346  C 0.106997 3.054567 -0.466325  C -0.106512 1.769667 -2.789685  H 0.613686 2.197725 -3.485829  H 0.102182 0.704150 -2.694334  H -1.105707 1.898003 -3.206006  C 0.259876 3.872011 0.735125  H 1.169877 3.599260 1.267968  H 0.309740 4.925513 0.460375  H -0.574949 3.717370 1.416182 | P 2.556822 -0.127616 0.275526  P -2.468916 0.089989 0.274987  Mg 0.121932 0.720559 0.128506  N -0.041915 -1.105323 -1.074023  C 2.281273 -1.631178 -0.591456  H 3.072973 -2.359742 -0.708780  C 1.052808 -1.946788 -1.155237  C 0.888552 -3.200837 -1.846891  H 1.742014 -3.861957 -1.909590  C -0.301550 -3.545659 -2.395003  H -0.409518 -4.493370 -2.907740  C -1.405606 -2.670740 -2.287478  H -2.370036 -2.921618 -2.702883  C -1.217198 -1.485604 -1.625854  C -2.375184 -0.532417 -1.461342  H -3.302092 -0.989590 -1.803242  H -2.193834 0.349750 -2.075735  C 3.931254 0.761623 -0.666541  C 5.065286 -0.173239 -1.098368  H 5.600497 -0.603023 -0.254403  H 5.787329 0.390160 -1.695986  H 4.686915 -0.987703 -1.715352  C 4.498389 1.940822 0.126766  H 3.706873 2.575136 0.529581  H 5.118180 2.555385 -0.531417  H 5.129507 1.610174 0.950964  C 3.250036 1.303043 -1.932000  H 2.741652 0.506866 -2.478537  H 4.003306 1.740128 -2.592760  H 2.520769 2.074778 -1.688565  C 3.109083 -0.565717 2.027109  C 4.462973 -1.274170 2.094510  H 4.499407 -2.136758 1.427516  H 4.641391 -1.631823 3.112403  H 5.282373 -0.603726 1.837781  C 3.121292 0.686861 2.913936  H 3.928971 1.369938 2.662194  H 3.258143 0.389123 3.957124  H 2.179773 1.233967 2.838845  C 2.027066 -1.508901 2.570534  H 1.053531 -1.015658 2.606795  H 2.281372 -1.798836 3.592999  H 1.930133 -2.408378 1.964216  C -2.982544 -1.411497 1.299189  C -1.715697 -2.253261 1.533981  H -1.324356 -2.684147 0.615274  H -1.962579 -3.075223 2.210295  H -0.924257 -1.666756 2.000062  C -4.031900 -2.302012 0.626810  H -4.963877 -1.779106 0.426263  H -4.259432 -3.142688 1.287121  H -3.657812 -2.717848 -0.308555  C -3.482098 -0.944708 2.672511  H -2.762052 -0.284270 3.158624  H -3.616173 -1.818091 3.314518  H -4.439400 -0.430050 2.618837  C -3.833414 1.390089 0.231696  C -5.255795 0.836571 0.115806  H -5.369808 0.198934 -0.761673  H -5.954243 1.670501 0.009316  H -5.558523 0.270278 0.994091  C -3.578571 2.301647 -0.979720  H -2.553070 2.668129 -1.005079  H -4.242110 3.166234 -0.908122  H -3.799370 1.800445 -1.921839  C -3.692488 2.250779 1.497006  H -3.885536 1.689478 2.408728  H -4.411734 3.072358 1.456064  H -2.690091 2.675119 1.568085  H 0.109683 2.176749 1.229393  C -0.045451 2.397253 -1.375758  C 0.053604 3.078846 -0.347740  C -0.158398 2.197451 -2.836052  H 0.148566 1.196332 -3.140161  H -1.198775 2.329212 -3.141808  H 0.440118 2.924536 -3.387968  C 0.158278 4.362262 0.374413  H 1.074587 4.399205 0.961236  H 0.157899 5.182524 -0.347843  H -0.679666 4.486760 1.059144 |
| **B** | **C** |
| P 2.513947 -0.253722 0.288172  P -2.400039 -0.024141 0.304050  Mg 0.170798 0.726529 -0.164911  N -0.030715 -1.076894 -1.257903  C 2.244975 -1.689421 -0.698046  H 3.033116 -2.419335 -0.829273  C 1.037835 -1.946122 -1.335095  C 0.852273 -3.153619 -2.098422  H 1.688850 -3.831829 -2.195164  C -0.346321 -3.440537 -2.665373  H -0.470708 -4.355863 -3.230411  C -1.441984 -2.561191 -2.502150  H -2.415350 -2.785706 -2.911417  C -1.226706 -1.411709 -1.791040  C -2.349170 -0.445484 -1.507092  H -3.300346 -0.829264 -1.871331  H -2.151898 0.495002 -2.024910  C 3.896410 0.704633 -0.570967  C 5.031289 -0.197537 -1.064876  H 5.561722 -0.689056 -0.251742  H 5.756796 0.408164 -1.614585  H 4.655879 -0.963560 -1.742525  C 4.461276 1.821533 0.309037  H 3.669983 2.431999 0.747428  H 5.086596 2.478117 -0.301136  H 5.087784 1.431418 1.110494  C 3.217755 1.342297 -1.793631  H 2.716931 0.590122 -2.406454  H 3.972267 1.832607 -2.413893  H 2.485458 2.092713 -1.493829  C 3.034194 -0.817117 2.010076  C 4.376173 -1.550967 2.035461  H 4.403138 -2.364893 1.309797  H 4.541075 -1.982205 3.026472  H 5.207516 -0.877552 1.830827  C 3.064006 0.370685 2.980938  H 3.876432 1.062831 2.776575  H 3.197364 -0.000317 4.000501  H 2.127900 0.931555 2.951556  C 1.929126 -1.770804 2.480445  H 0.970608 -1.252354 2.547006  H 2.171375 -2.142061 3.479076  H 1.810367 -2.620011 1.809041  C -2.909866 -1.636707 1.148639  C -1.654505 -2.520154 1.251161  H -1.300624 -2.857404 0.279862  H -1.899921 -3.406339 1.841453  H -0.837283 -2.003240 1.750379  C -3.988959 -2.427559 0.401631  H -4.920330 -1.877942 0.293265  H -4.207330 -3.340508 0.961351  H -3.645609 -2.730583 -0.586963  C -3.374271 -1.331329 2.578689  H -2.633226 -0.746878 3.126994  H -3.512342 -2.273563 3.113425  H -4.322537 -0.798449 2.607818  C -3.765683 1.272260 0.427239  C -5.191307 0.736059 0.276681  H -5.322374 0.194041 -0.660470  H -5.886258 1.579407 0.267444  H -5.484351 0.083419 1.096609  C -3.524860 2.312392 -0.678680  H -2.498370 2.678111 -0.674269  H -4.184189 3.164775 -0.500120  H -3.761958 1.920525 -1.667381  C -3.605842 1.991066 1.776059  H -3.746314 1.328269 2.627526  H -4.351807 2.785951 1.849341  H -2.619507 2.450092 1.855440  C 0.002664 2.750520 -0.788460  C 0.097070 3.698035 0.156689  C -0.184040 3.112052 -2.247158  H -0.179478 4.181344 -2.478496  H 0.593958 2.644343 -2.857959  H -1.131141 2.707913 -2.621310  C 0.039033 5.201596 0.030953  H 0.942766 5.661415 0.440021  H -0.068402 5.540870 -0.997533  H -0.802218 5.604948 0.602205  H 0.229398 3.379596 1.193937 | P 2.522326 -0.257613 0.268404  P -2.407181 -0.018866 0.268419  Mg 0.172884 0.707860 -0.192708  N -0.029984 -1.120855 -1.248578  C 2.246529 -1.720280 -0.675582  H 3.032579 -2.455417 -0.788556  C 1.038695 -1.991753 -1.304830  C 0.853358 -3.217989 -2.037998  H 1.689624 -3.899012 -2.115862  C -0.343645 -3.517329 -2.601522  H -0.467577 -4.446050 -3.144379  C -1.437997 -2.631938 -2.464552  H -2.409805 -2.863772 -2.873502  C -1.223688 -1.465952 -1.780371  C -2.346144 -0.490688 -1.529240  H -3.295391 -0.883014 -1.889450  H -2.143854 0.435335 -2.070766  C 3.915945 0.662888 -0.611478  C 5.045334 -0.263346 -1.071645  H 5.562248 -0.738905 -0.240199  H 5.782831 0.319925 -1.629653  H 4.668346 -1.043136 -1.732443  C 4.487312 1.799371 0.238831  H 3.700158 2.421520 0.668081  H 5.110815 2.440063 -0.389805  H 5.117446 1.425575 1.045076  C 3.247498 1.270653 -1.854782  H 2.743497 0.505271 -2.448266  H 4.008215 1.738239 -2.484971  H 2.519149 2.033862 -1.578366  C 3.022545 -0.779444 2.008532  C 4.321892 -1.584607 2.058432  H 4.302073 -2.423817 1.362028  H 4.467339 -1.988677 3.063928  H 5.187386 -0.964284 1.828332  C 3.121406 0.436663 2.938865  H 3.997556 1.047742 2.739919  H 3.189710 0.093499 3.974443  H 2.240862 1.076094 2.857022  C 1.864108 -1.652547 2.508407  H 0.936496 -1.077720 2.555649  H 2.084285 -2.005663 3.518681  H 1.698624 -2.514967 1.864009  C -2.928545 -1.604667 1.154989  C -1.672834 -2.481592 1.298448  H -1.304505 -2.842942 0.341214  H -1.923590 -3.352669 1.908691  H -0.864825 -1.947611 1.794754  C -3.998394 -2.417469 0.418733  H -4.927308 -1.870092 0.280719  H -4.225877 -3.312302 1.003591  H -3.642246 -2.751510 -0.555210  C -3.409860 -1.258885 2.570210  H -2.673111 -0.663961 3.112690  H -3.558834 -2.186193 3.127758  H -4.356086 -0.721645 2.573100  C -3.769181 1.284619 0.344954  C -5.195033 0.749123 0.195196  H -5.318907 0.183285 -0.728786  H -5.887571 1.593719 0.157677  H -5.497418 0.118479 1.028809  C -3.513685 2.295000 -0.784816  H -2.485420 2.655725 -0.779646  H -4.170090 3.154990 -0.633940  H -3.743544 1.879687 -1.765613  C -3.617713 2.039067 1.675203  H -3.751249 1.397897 2.544245  H -4.371167 2.828504 1.728015  H -2.636349 2.510676 1.742017  C 0.027051 2.721426 -0.854731  C 0.142924 3.677801 0.079390  C -0.157179 3.068496 -2.317087  H -0.156505 4.135665 -2.558356  H 0.625678 2.598868 -2.920283  H -1.100381 2.656166 -2.691720  C 0.112844 5.180833 -0.062605  H 1.032546 5.626517 0.326211  H -0.004761 5.511272 -1.092871  H -0.710074 5.607525 0.518225  H 0.276295 3.367819 1.118880  H -0.763599 1.203302 2.818709  H -0.176868 1.240262 3.276660 |
| **TS2** | **Mg-Pincer** |
| P -2.421578 -0.502176 -0.336189  P 2.528374 0.086359 -0.400150  Mg -0.104349 0.636303 -0.373645  N 0.178848 -0.592970 1.363883  C -2.061875 -1.481901 1.079425  H -2.815552 -2.143910 1.485452  C -0.855223 -1.418338 1.761661  C -0.640882 -2.250927 2.917880  H -1.455872 -2.878076 3.251925  C 0.554881 -2.263507 3.554947  H 0.703632 -2.901635 4.417003  C 1.614703 -1.454926 3.084670  H 2.585941 -1.464027 3.555844  C 1.371200 -0.651607 2.003487  C 2.472400 0.214944 1.443883  H 3.424442 -0.020631 1.915306  H 2.246837 1.258093 1.661914  C -3.916191 0.562789 0.108443  C -5.192910 -0.249677 0.335067  H -5.582890 -0.662010 -0.594846  H -5.966310 0.400618 0.752613  H -5.032893 -1.067824 1.038546  C -4.165387 1.634349 -0.959486  H -3.258015 2.194064 -1.178954  H -4.915163 2.340450 -0.592557  H -4.540829 1.217092 -1.890692  C -3.539279 1.265708 1.421688  H -3.399328 0.549239 2.229426  H -4.342725 1.952336 1.700178  H -2.623211 1.847716 1.317927  C -2.838573 -1.711588 -1.724063  C -3.724559 -2.876157 -1.270394  H -3.266532 -3.417522 -0.443120  H -3.848897 -3.577264 -2.100088  H -4.715950 -2.552846 -0.961922  C -3.474197 -1.003474 -2.922402  H -4.504119 -0.711875 -2.721495  H -3.489553 -1.683350 -3.777975  H -2.913676 -0.113328 -3.213685  C -1.484232 -2.290753 -2.152221  H -0.828615 -1.520320 -2.564933  H -1.633759 -3.047437 -2.926287  H -0.981862 -2.765481 -1.308874  C 3.181292 -1.656169 -0.723895  C 1.978350 -2.607885 -0.617123  H 1.548935 -2.629914 0.382374  H 2.307161 -3.620690 -0.862311  H 1.194895 -2.330899 -1.321316  C 4.254048 -2.110675 0.270866  H 5.125280 -1.459184 0.278163  H 4.591557 -3.112424 -0.006267  H 3.857243 -2.172398 1.283749  C 3.720010 -1.757136 -2.156050  H 2.994288 -1.399615 -2.888165  H 3.925726 -2.806120 -2.380011  H 4.648858 -1.206919 -2.292694  C 3.772037 1.399963 -0.920800  C 5.222150 1.102934 -0.532270  H 5.326486 0.929625 0.539577  H 5.844157 1.964299 -0.788351  H 5.626577 0.241625 -1.060748  C 3.349110 2.718352 -0.252220  H 2.294518 2.942474 -0.412823  H 3.931055 3.531907 -0.690071  H 3.548531 2.718087 0.818888  C 3.651579 1.599492 -2.439868  H 3.971130 0.728181 -3.006209  H 4.285732 2.437004 -2.739932  H 2.624107 1.835297 -2.719714  C -0.271538 2.861914 0.302856  C -1.238797 3.610648 -0.231644  C 0.194078 3.024490 1.730538  H 0.072490 2.096295 2.295912  H 1.258376 3.270299 1.759464  H -0.329660 3.810252 2.280500  C -2.103519 4.666771 0.388766  H -2.009259 5.612494 -0.149765  H -3.153892 4.369790 0.322218  H -1.874580 4.847645 1.437282  H -1.454801 3.432885 -1.289802  H 0.214616 1.891015 -1.891133  H 0.211633 2.333526 -0.978339 | P -2.519544 -0.310620 -0.005677  P 2.411045 -0.492984 -0.142367  Mg -0.167744 -0.810481 -0.939637  N 0.027791 1.295590 -0.681380  C -2.253980 1.424816 0.124127  H -3.041573 2.074942 0.481904  C -1.043180 2.021619 -0.200369  C -0.859079 3.439953 -0.029064  H -1.698002 4.025436 0.320969  C 0.338972 4.022152 -0.283818  H 0.462223 5.088784 -0.143797  C 1.434961 3.237452 -0.710989  H 2.407854 3.670466 -0.887764  C 1.222042 1.896658 -0.884676  C 2.346928 0.974960 -1.280096  H 3.295016 1.507507 -1.325425  H 2.146164 0.573970 -2.274695  C -3.898875 -0.525413 -1.277091  C -5.038502 0.482197 -1.098384  H -5.571510 0.350660 -0.158919  H -5.760812 0.353919 -1.909072  H -4.666958 1.505438 -1.142393  C -4.458109 -1.949445 -1.289463  H -3.664979 -2.697934 -1.329712  H -5.084955 -2.082684 -2.174740  H -5.081574 -2.149152 -0.418480  C -3.219127 -0.249351 -2.627739  H -2.738368 0.730569 -2.634510  H -3.969683 -0.260978 -3.421998  H -2.468797 -1.005869 -2.863624  C -3.036930 -0.947466 1.691214  C -4.385145 -0.402447 2.166076  H -4.423685 0.685858 2.104161  H -4.547962 -0.682887 3.210156  H -5.211795 -0.811294 1.586362  C -3.052478 -2.481568 1.709681  H -3.854312 -2.901926 1.108324  H -3.195120 -2.828733 2.736355  H -2.108512 -2.893361 1.347885  C -1.938182 -0.481501 2.654824  H -0.970048 -0.903067 2.377259  H -2.170417 -0.828747 3.664420  H -1.842779 0.603159 2.669322  C 2.957848 0.224128 1.517687  C 1.712117 0.830243 2.186718  H 1.315429 1.681998 1.638944  H 1.986467 1.175813 3.186414  H 0.917202 0.094391 2.295875  C 4.024569 1.318301 1.405625  H 4.938839 0.970061 0.931255  H 4.283318 1.663462 2.409736  H 3.652135 2.180795 0.853955  C 3.461330 -0.908580 2.421280  H 2.735746 -1.720559 2.494241  H 3.616494 -0.513662 3.427651  H 4.409453 -1.322997 2.084112  C 3.747094 -1.585922 -0.898419  C 5.178837 -1.067488 -0.745035  H 5.288401 -0.058653 -1.144389  H 5.853683 -1.717937 -1.307072  H 5.515396 -1.066812 0.290083  C 3.434794 -1.714352 -2.399535  H 2.386275 -1.953688 -2.582660  H 4.043193 -2.521828 -2.812666  H 3.687208 -0.806081 -2.946033  C 3.619162 -2.985803 -0.277831  H 3.819113 -2.993644 0.791459  H 4.339721 -3.655383 -0.753153  H 2.622344 -3.396072 -0.443730  H 0.025639 -1.891421 -2.318375 |
| **D** |  |
| P 2.528908 0.307101 -0.051361  P -2.583686 -0.171843 0.068967  Mg 0.048083 -0.221271 -0.735409  N -0.164456 1.714369 0.164453  C 2.167792 2.017643 -0.344300  H 2.961546 2.711459 -0.588070  C 0.900425 2.536314 -0.165899  C 0.634707 3.947936 -0.300189  H 1.424488 4.587083 -0.670513  C -0.565386 4.457470 0.068838  H -0.749597 5.521426 -0.013867  C -1.575871 3.606111 0.581388  H -2.515090 3.996107 0.943733  C -1.322429 2.261011 0.594750  C -2.328594 1.292646 1.161272  H -3.261860 1.795598 1.405236  H -1.911960 0.903458 2.090197  C 3.185740 0.224136 1.726157  C 4.014136 1.454880 2.102910  H 4.934851 1.523761 1.525720  H 4.287829 1.398586 3.160198  H 3.445759 2.370768 1.946026  C 3.991850 -1.044422 2.009715  H 3.470754 -1.943023 1.682694  H 4.160397 -1.131522 3.086508  H 4.969095 -1.023746 1.529236  C 1.916214 0.207158 2.593668  H 1.280391 1.068900 2.387280  H 2.191786 0.232289 3.651383  H 1.330131 -0.698735 2.421146  C 3.872152 -0.135944 -1.289859  C 5.207609 0.567231 -1.034043  H 5.089015 1.649737 -0.975871  H 5.893147 0.353576 -1.858589  H 5.684448 0.225377 -0.116357  C 4.071415 -1.657224 -1.309263  H 4.486572 -2.040007 -0.379481  H 4.763529 -1.922529 -2.112675  H 3.127359 -2.167677 -1.499576  C 3.343495 0.285175 -2.669440  H 2.370779 -0.161430 -2.880285  H 4.048577 -0.048585 -3.435410  H 3.237656 1.366143 -2.745934  C -3.668012 0.504511 -1.322631  C -2.721171 1.238973 -2.288646  H -2.220843 2.081808 -1.811700  H -3.310028 1.633929 -3.120315  H -1.960935 0.572114 -2.695234  C -4.744172 1.494650 -0.864937  H -5.430554 1.074569 -0.133584  H -5.332487 1.797549 -1.734548  H -4.301605 2.398689 -0.447534  C -4.316018 -0.656227 -2.085160  H -3.582115 -1.410824 -2.372730  H -4.766540 -0.269015 -3.001676  H -5.107120 -1.136796 -1.510930  C -3.530559 -1.372621 1.172800  C -4.947086 -0.924470 1.541741  H -4.958530 0.073558 1.980479  H -5.360286 -1.615145 2.281209  H -5.614064 -0.931982 0.681265  C -2.715443 -1.527604 2.468986  H -1.647649 -1.650596 2.278431  H -3.060792 -2.415352 3.002200  H -2.843906 -0.676434 3.135980  C -3.590493 -2.745034 0.485815  H -4.153744 -2.724283 -0.443580  H -4.082907 -3.456012 1.153362  H -2.596017 -3.131427 0.267902  H 0.006914 -0.656152 -2.437363  C 0.005126 -3.357841 -0.954499  C 0.246540 -3.365215 0.355503  C 0.918709 -3.796654 -2.052973  C 1.493120 -3.824612 1.048538  H 0.454350 -4.603343 -2.623990  H 1.085571 -2.967897 -2.743753  H 1.879911 -4.154213 -1.689178  H 1.707969 -3.189846 1.908182  H 1.368527 -4.841038 1.429811  H 2.363988 -3.815689 0.395260  H -0.556226 -3.047220 1.012667  H -0.967396 -3.008027 -1.285147 |  |

**Table S9.** Cartesian coordinates of stationary points of hydrogenation of 2-butyne in the gas phase using CAl_3_MgH_2_^¯^ catalyst at ωB97XD/6-311++G(2d,2p) level of theory.

| **A** | **TS1** |
| --- | --- |
| C 2.952446 -0.153977 0.299839  C 2.110060 -0.821825 0.839596  Mg 0.569424 0.467786 -1.264614  C -1.267120 0.337340 -0.227282  Al -1.191514 1.959307 0.779694  Al -1.500117 -1.337206 -1.223642  Al -3.062604 -0.030706 0.531965  H 1.731185 1.227630 -2.317935  H -3.093192 -1.555836 -0.464348  C 4.018449 0.636340 -0.310842  C 1.148688 -1.649654 1.563589  H 4.900117 0.018264 -0.483750  H 3.675842 1.043364 -1.263427  H 4.297314 1.466102 0.339131  H 1.600876 -2.035774 2.477998  H 0.261096 -1.067324 1.808578  H 0.823597 -2.487327 0.945489 | C 3.560965 0.121996 -0.126006  C 2.946460 -0.068970 0.935209  Mg 1.248230 -0.225593 -0.514190  C -0.775768 0.138239 -0.038406  Al -0.759062 2.029638 0.194287  Al -0.899218 -1.820736 -0.159565  Al -2.719478 0.084757 0.354743  H 2.628398 0.034537 -1.629921  H -2.627261 -1.743715 0.191404  C 4.793155 0.420023 -0.887035  C 2.869176 -0.164073 2.407363  H 5.625691 0.537097 -0.187598  H 5.019565 -0.385767 -1.584005  H 4.675812 1.337158 -1.462542  H 3.829581 0.023963 2.895679  H 2.138283 0.548810 2.790662  H 2.517540 -1.153636 2.701214 |
| **B** | **C** |
| C 3.210693 -0.786253 -0.000382  C 2.382300 0.269338 0.000199  Mg 0.317113 -0.164524 0.000049  C -1.769409 0.071540 0.000138  Al -1.920792 1.973360 0.000040  Al -1.729043 -1.895358 0.000885  Al -3.749926 -0.119919 -0.000863  H 2.774665 -1.785971 -0.000715  H -3.485965 -1.947832 0.000021  C 4.720723 -0.821397 -0.000679  C 2.904419 1.690499 0.000659  H 5.173744 0.168401 -0.000006  H 5.093797 -1.358669 0.876125  H 5.093432 -1.357256 -0.878507  H 3.994637 1.789513 0.002511  H 2.528944 2.233985 -0.870922  H 2.525918 2.234676 0.870475 | C 3.205435 -0.810077 -0.131250  C 2.370706 0.237873 -0.060104  Mg 0.303090 -0.180562 0.005357  C -1.784366 0.056946 -0.045199  Al -1.935409 1.956073 -0.138702  Al -1.753393 -1.902150 0.110759  Al -3.761134 -0.132168 -0.141397  H 2.775280 -1.811655 -0.166829  H -3.509377 -1.952349 0.015335  C 4.715107 -0.833554 -0.174717  C 2.884832 1.661129 -0.006469  H 5.161528 0.158275 -0.128912  H 5.116491 -1.418842 0.657736  H 5.066486 -1.313337 -1.092932  H 3.973802 1.767170 0.029616  H 2.530037 2.224626 -0.874177  H 2.478634 2.182323 0.865582  H 1.002217 0.689477 2.625367  H 0.266714 0.784329 2.712786 |
| **TS2** | **D** |
| C 3.091847 -0.831032 0.596120  C 2.394966 0.299175 0.750431  Mg 0.316030 -0.509890 0.951173  C -1.559169 -0.181787 0.079193  Al -1.773686 1.693471 0.360233  Al -1.448451 -2.116029 -0.265951  Al -3.320272 -0.273836 -0.834872  H 2.790065 -1.686715 1.213956  H -3.016211 -2.076371 -1.065178  C 4.264210 -1.126422 -0.296485  C 2.710172 1.592040 0.049936  H 4.567064 -0.261874 -0.885335  H 5.124820 -1.461502 0.288770  H 4.017503 -1.934965 -0.988728  H 3.683690 1.612666 -0.451141  H 1.945791 1.822087 -0.697157  H 2.685373 2.418663 0.763327  H 1.668290 0.019026 2.068428  H 1.123971 -0.459467 2.737454 | C 2.451802 -0.723655 -0.519615  C 1.999529 0.461133 -0.934936  Mg 0.301467 -0.210072 1.358053  C -1.475734 -0.201918 0.215347  Al -1.534987 -2.026406 -0.339270  Al -1.483818 1.721156 0.593256  Al -3.237142 0.104933 -0.646736  H 1.431705 -0.724109 2.578757  H -3.066617 1.865931 -0.197479  H 1.052501 0.468875 -1.464740  H 1.841271 -1.592354 -0.756852  C 2.642477 1.801318 -0.750724  C 3.710483 -1.014109 0.236560  H 2.897216 2.232384 -1.722079  H 1.939982 2.486708 -0.272010  H 3.546943 1.760636 -0.146811  H 4.304242 -1.763099 -0.292055  H 4.328066 -0.129692 0.381625  H 3.463050 -1.416922 1.220969 |

**Table S10.** Cartesian coordinates of stationary points of hydrogenation of 2-butene in PCM solvation of toluene using CAl_3_MgH_2_^¯^ catalyst at ωB97XD/6-311++G(2d,2p) level of theory.

| **A** | **TS1** |
| --- | --- |
| C -2.088197 0.627961 -0.850141  C -2.361184 -0.677884 -0.781848  Mg -0.381942 -0.284240 1.235361  C 1.463310 0.048108 0.239083  Al 1.511348 1.959076 0.247536  Al 1.538892 -1.894794 0.002158  Al 3.264964 0.068585 -0.582391  H -1.602840 -0.210029 2.487908  H 3.172943 -1.726031 -0.711871  H -1.714235 -1.345122 -1.343564  H -1.239451 0.919752 -1.460674  C -3.481594 -1.342278 -0.043115  C -2.828956 1.753546 -0.195480  H -4.173489 -1.800992 -0.753546  H -3.099226 -2.142813 0.592304  H -4.040331 -0.653884 0.586180  H -3.353308 2.344865 -0.949952  H -3.553545 1.411742 0.540053  H -2.131152 2.429411 0.302909 | C 2.780469 0.844397 -0.148925  C 2.327723 0.125921 0.967187  Mg 0.476436 0.171683 -0.349376  C -1.623773 0.140628 -0.089888  Al -1.959560 1.985105 0.261115  Al -1.479002 -1.754104 -0.587566  Al -3.547771 -0.235946 0.192249  H 1.747749 0.903286 -1.440439  H -3.225359 -1.954130 -0.289715  H 1.981662 0.705559 1.816687  H 2.733982 1.924096 -0.062481  C 2.717451 -1.316024 1.217454  C 3.924067 0.351497 -1.001493  H 3.775149 -1.434903 1.482191  H 2.128366 -1.736761 2.032243  H 2.540400 -1.958167 0.348251  H 4.847241 0.418654 -0.418248  H 3.790796 -0.689220 -1.291104  H 4.029484 0.947165 -1.906270 |
| **B** | **C** |
| C -3.243137 -0.843123 -0.169906  C -2.358287 0.414375 -0.152073  Mg -0.273017 -0.048620 -0.056340  C 1.842230 0.056155 -0.044261  Al 2.090066 1.926504 -0.353464  Al 1.780705 -1.881759 0.263422  Al 3.816791 -0.153959 -0.003465  H -3.156004 -1.363399 0.791606  H 3.566307 -1.922385 0.281436  C -4.729286 -0.601649 -0.459755  C -2.789633 1.398714 0.946827  H -4.853463 -0.088794 -1.416877  H -5.286000 -1.540742 -0.507395  H -5.195699 0.017046 0.308471  H -3.827623 1.742676 0.847757  H -2.704633 0.944639 1.939947  H -2.165033 2.295692 0.955108  H -2.866017 -1.550475 -0.915787  H -2.505249 0.922140 -1.117605 | C 3.182425 -0.796606 0.332882  C 2.351158 0.209077 -0.481169  Mg 0.249826 -0.116778 -0.253450  C -1.854022 0.037074 -0.054888  Al -2.053010 1.936780 0.028727  Al -1.848875 -1.925578 -0.070915  Al -3.831077 -0.123159 0.059983  H 2.812031 -1.810938 0.152751  H -3.632936 -1.915694 0.034415  C 4.690218 -0.789366 0.054806  C 2.780496 1.658541 -0.207974  H 4.883350 -0.967921 -1.005968  H 5.145658 0.167020 0.316304  H 5.206095 -1.565808 0.624820  H 3.834822 1.857149 -0.441028  H 2.192213 2.372328 -0.789821  H 2.638466 1.922749 0.845372  H 1.337134 0.733557 2.566762  H 0.624572 0.765099 2.783018  H 2.558222 0.003738 -1.542815  H 3.029312 -0.616834 1.404310 |
| **TS2** | **D** |
| C 3.252784 -0.636585 0.206085  C 2.336516 0.586551 0.263163  Mg 0.220293 -0.103262 0.886356  C -1.745771 0.033099 0.154342  Al -2.042155 1.918909 0.225868  Al -1.569703 -1.910599 -0.066128  Al -3.604792 -0.221065 -0.482374  H 3.383272 -1.034063 1.220856  H -3.248683 -2.005492 -0.622319  C 4.637154 -0.389296 -0.396275  C 1.874415 1.052204 -1.129042  H 5.167148 0.380808 0.168889  H 5.248226 -1.294392 -0.386412  H 4.566860 -0.050180 -1.431222  H 2.712499 1.340646 -1.774444  H 1.326209 0.268501 -1.662946  H 1.210257 1.918484 -1.070649  H 1.719592 0.427816 1.653012  H 1.297431 0.195799 2.524810  H 2.759121 -1.434628 -0.361329  H 2.900410 1.415815 0.710094 | C -2.722217 0.815758 -0.160441  C -2.160244 -0.356016 -0.965441  Mg 0.403041 -0.525424 1.818366  C 1.656608 0.163192 0.267729  Al 1.366281 2.051711 0.394155  Al 2.008200 -1.733665 -0.091436  Al 2.995048 0.521498 -1.151752  H -0.755391 -0.619951 3.119666  H 3.192804 -1.262025 -1.345361  H -1.071808 -0.265645 -1.000860  H -2.304013 1.746471 -0.551080  C -2.532115 -1.725969 -0.404059  C -4.245870 0.902541 -0.170108  H -3.605108 -1.914192 -0.468729  H -2.021070 -2.523378 -0.944262  H -2.251055 -1.806753 0.649693  H -4.593393 1.781716 0.373649  H -4.624664 0.969505 -1.192765  H -4.700603 0.027317 0.296055  H -2.376409 0.739975 0.875714  H -2.506643 -0.279060 -2.000756 |

**Table S11.** Cartesian coordinates of CAl_3_MgH_2_^¯^ in gas phase and in PCM solvation of toluene at ωB97XD/6-311++G(2d,2p) level of theory.

| **CAl_3_MgH_2_**^¯^ | |
| --- | --- |
| **Solvent phase** | **Gas phase** |
| Mg 2.090918 0.399393 0.000000  C -0.001838 0.110755 0.000000  Al -0.610914 1.927812 0.000000  Al 0.409568 -1.810126 0.000000  Al -1.905705 -0.454665 0.000000  H 3.709941 1.054848 0.000000  H -1.346279 -2.166260 0.000000 | Mg 2.094762 0.016680 0.000000  C 0.000000 0.129739 0.000000  Al -0.259917 2.023625 0.000000  Al 0.130436 -1.832799 0.000000  Al -1.968935 -0.151088 0.000000  H 3.769626 0.462847 0.000000  H -1.627362 -1.958032 0.000000 |
